# Increased expression of RUNX3 inhibits normal human myeloid development

**DOI:** 10.1038/s41375-022-01577-2

**Published:** 2022-04-30

**Authors:** Ana Catarina Menezes, Rachel Jones, Alina Shrestha, Rachael Nicholson, Adam Leckenby, Aleksandra Azevedo, Sara Davies, Sarah Baker, Amanda F. Gilkes, Richard L. Darley, Alex Tonks

**Affiliations:** 1 Department of Haematology, Division of Cancer & Genetics, School of Medicine, Cardiff University, Cardiff, CF14 4XN UK; 2 Cardiff Experimental Cancer Medicine Centre (ECMC), School of Medicine, Cardiff University, Cardiff, CF14 4XN UK

**Keywords:** Acute myeloid leukaemia, Haematopoiesis, Haematopoietic stem cells, Differentiation

## Abstract

RUNX3 is a transcription factor dysregulated in acute myeloid leukemia (AML). However, its role in normal myeloid development and leukemia is poorly understood. Here we investigate RUNX3 expression in both settings and the impact of its dysregulation on myelopoiesis. We found that *RUNX3* mRNA expression was stable during hematopoiesis but decreased with granulocytic differentiation. In AML, *RUNX3* mRNA was overexpressed in many disease subtypes, but downregulated in AML with core binding factor abnormalities, such as RUNX1::ETO. Overexpression of RUNX3 in human hematopoietic stem and progenitor cells (HSPC) inhibited myeloid differentiation, particularly of the granulocytic lineage. Proliferation and myeloid colony formation were also inhibited. Conversely, RUNX3 knockdown did not impact the myeloid growth and development of human HSPC. Overexpression of RUNX3 in the context of RUNX1::ETO did not rescue the RUNX1::ETO-mediated block in differentiation. RNA-sequencing showed that RUNX3 overexpression downregulates key developmental genes, such as *KIT* and *RUNX1*, while upregulating lymphoid genes, such as *KLRB1* and *TBX21*. Overall, these data show that increased RUNX3 expression observed in AML could contribute to the developmental arrest characteristic of this disease, possibly by driving a competing transcriptional program favoring a lymphoid fate.

## Introduction

Acute myeloid leukemia (AML) is an aggressive bone marrow (BM) malignancy, presenting with a clonal expansion of immature myeloid cells coupled with a block in differentiation. The complexity and heterogeneity of AML is associated with the presence of different molecular abnormalities. *RUNX1* is one of the most altered genes identified in AML [1], and together with its family members RUNX2 and RUNX3, plays an important role in the regulation of different developmental processes, such as hematopoiesis [2]. Accounting for 12% of all cases, t(8; 21)(q22; q22.1) is a common chromosomal abnormality observed in AML patients that generates the RUNX1::ETO fusion oncogene [3] and has been associated with the downregulation of *RUNX3* [4].

RUNX3 is one of three mammalian Runt-domain transcription factors (TF) and was initially cloned based on its similarity to RUNX1 [5]. Genetically engineered mouse models lacking RUNX3 have demonstrated the importance of this TF in a variety of physiological processes, including neurogenesis, thymopoiesis, and dendritic cell functional maturation [6–8]. Previously linked to leukemogenesis, RUNX3 overexpression in BCR::ABL cells was shown to protect these cells from Imatinib-induced apoptosis, involving RUNX3 in chronic myeloid leukemia persistence [9]. In core binding factor (CBF) AML comprising t(8;21) and inv(16) subtypes, lower *RUNX3* expression is observed [4, 10]. Expression of RUNX3 has been associated with chemoresistance of leukemic cells and shortened event free survival (EFS) and overall survival (OS) of childhood AML patients [4]. Here we have investigated *RUNX3* expression in normal hematopoiesis and AML, and further determined its role in myelopoiesis. This study suggests that RUNX3 is an important regulator of human hematopoiesis, and its overexpression might contribute to the pathogenesis of AML.

## Methods

### Plasmids and generation of retro- and lentivirus

A retroviral vector co-expressing RUNX3 and *Discosoma* sp. red fluorescent protein (DsRed) was generated by directional cloning of *RUNX3* (NM_001031680.2) into *Bam*H1/*Eco*R1 sites of a PINCO vector modified to express DsRed [11, 12]. PINCO co-expressing RUNX1::ETO and green fluorescent protein (GFP) was also employed in this study [13]. PINCO DsRed/GFP vectors lacking *RUNX3* or *RUNX1::ETO* cDNA were used as controls. Short hairpin RNA (shRNA) vectors co-expressing GFP were purchased from VectorBuilder (Guangzhou, China) (Supplemental Materials and Methods). Retro- and lentivirus were subsequently generated by transient transfection of Phoenix or HEK293T packaging cells, respectively, using Lipofectamine 3000 (Fisher Scientific, Loughborough, UK) according to manufacturer’s instructions.

### Generation of control and RUNX3 expressing/knockdown human myeloid progenitor cells

Human neonatal cord blood was obtained from the Maternity Unit of the University Hospital of Wales (Cardiff) in accordance with the 1964 Declaration of Helsinki. Human CD34^+^ HSPC were isolated, cultured and transduced with retro/lentivirus as previously described (Supplemental Materials and Methods) [12, 13]. Following infection (day 3 of culture), cells were maintained in bulk liquid culture for growth and differentiation assessment by flow cytometry (as below).

### Phenotypic, differentiation, migration, and morphological analysis

To assess myeloid cell growth and differentiation in bulk liquid culture, human HSPC were maintained in Iscove’s Modified Dulbecco’s Medium (IMDM; Fisher Scientific, Loughborough, UK) supplemented with IL-3, SCF, G-CSF and GM-CSF (BioLegend, London, UK) at 5 ng/mL for 13 days. Transduced cultures were analyzed by flow cytometry for cell surface markers expression using a BD FACSCanto™II (Supplemental Materials and Methods) as previously described [12, 14]. The gating strategy employed in these studies is shown in Supplemental Fig. S1. Granulocytic cells were defined as CD13^–/+^CD36^–^, monocytic cells as CD13^+^CD36^+^, and erythroid cells as CD13^–^CD36^+^. Measurement of cell motility using the Transwell^®^ cell migration assay was performed on day 6 of culture using stromal cell-derived factor 1 (SDF-1) as a chemoattractant (Supplemental Materials and Methods). Morphology was assessed on day 17 of culture (Supplemental Materials and Methods) [13].

### Colony assay

Myeloid colony assays were performed on day 3 using HSPC FACSorted for DsRed or GFP positivity (or both in case of double transduced cells expressing RUNX3 and RUNX1::ETO) using a BD FACSAria™III (BD Biosciences, Wokingham, UK). Sorted HSPC were plated by limiting dilution in 96-U plates (0.3 cells/well) in IMDM supplemented with IL-3, SCF, G-CSF, and GM-CSF at 5 ng/mL and incubated at 37 °C with 5% CO_2_ [12]. Following 7 days of growth, individual myeloid colonies (>50 cells) and clusters (>5, <50 cells) were counted and scored. To assess their self-renewal potential, colonies were harvested, replated at higher density (1 cell/well), and cultured for an additional week.

For erythroid colony assays, HSPC co-expressing RUNX1::ETO and RUNX3 and respective controls were sorted for both GFP and DsRed positivity on day 3 of culture. Briefly, cultures were pre-stained with CD13-allophycocyanin (APC) for 30 min at RT to enrich for erythroid-committed cells (GFP^+^DsRed^+^, CD13^–^). Cells were index sorted into 96-U plates containing IMDM supplemented with IL-3, IL-6, SCF (5 ng/mL) and erythropoietin (EPO, 2 U/mL) at a density of 1 cell/well and incubated at 37 °C, 5% CO_2_. Colonies and clusters were counted and scored after one week of growth.

### Validation of RUNX3 expression by western blot and qRT-PCR

Cytosolic and nuclear proteins were extracted using the Biovision Nuclear/Cytosol Fractionation Kit (Cambridge Bioscience, Cambridge, UK) following manufacturer’s instructions. Western blotting was performed as previously described (Supplemental Material and Methods) [15] and RUNX3 protein expression was detected using a primary rabbit monoclonal antibody (D6E2, Cell Signaling Technologies, London, UK).

Total RNA was extracted from GFP^+^ sorted HSPC using the RNeasy Plus Mini Kit (Qiagen, Manchester, UK) and *RUNX3* mRNA expression was determined using a TaqMan gene expression assay (Hs00231709_m1, Fisher Scientific UK Ltd, Loughborough, UK) (Supplemental Materials and Methods).

### RNA sequencing

RNA-sequencing was performed by Novogene (Cambridge, UK) to identify early transcriptomic changes caused by overexpression of RUNX3 in human primary HSPC (Supplemental Materials and Methods).

### Statistical and data analysis

Statistical analysis was performed using a two-sided, equal variance paired sample t-test, or one-way ANOVA using Tukey’s multiple comparisons test. Minitab 18 software (Minitab LLC, State College, Pennsylvania, USA) was used for all statistical analyses.

For RNA-seq experiments, differential expression analysis was performed using DESeq2 (*n* = 5). Enrichment analysis of differentially expressed (DE) genes was performed using the Kyoto Encyclopedia of Genes and Genomes (KEGG) and Ingenuity^®^ Pathway Analysis (IPA^®^, QIAGEN, Manchester, UK). To reduce and filter the DE gene list for further analysis, a cut-off was established of fold changes ≥1.5 and an adjusted *p*-value (padj) < 0.05. RNA-seq data is deposited in GSE181059.

Gene expression data was obtained from GSE17054 (ref. [16]), GSE19599 (ref. [17]), GSE11864 (ref. [18]), GSE42519 (ref. [19, 20]), GSE13159 (ref. [21, 22]), E-MEXP-1242 (ref. [23]), TCGA dataset [24], and the Therapeutically Applicable Research to Generate Effective Treatments (TARGET, https://ocg.cancer.gov/programs/target, phs000465) initiative. TARGET data is available at https://portal.gdc.cancer.gov/projects. *RUNX3* mRNA expression in normal human hematopoietic cells and AML patient samples was analyzed using BloodSpot [25]. Batch correction between different datasets was performed by RMA normalization [25], with AML samples being compared to their closest normal counterpart following the method described by Rapin et al [19]. TCGA and TARGET AML datasets were analyzed using cBioPortal [24, 26, 27]. RUNX1::ETO HSPC dataset (E-MEXP-583) was used to assess *RUNX3* expression and to further compare RUNX3-mediated transcriptional dysregulation with RUNX1::ETO-associated expression changes using IPA^®^ [28].

## Results

### RUNX3 expression decreases during human neutrophil development

**Fig. 1:**
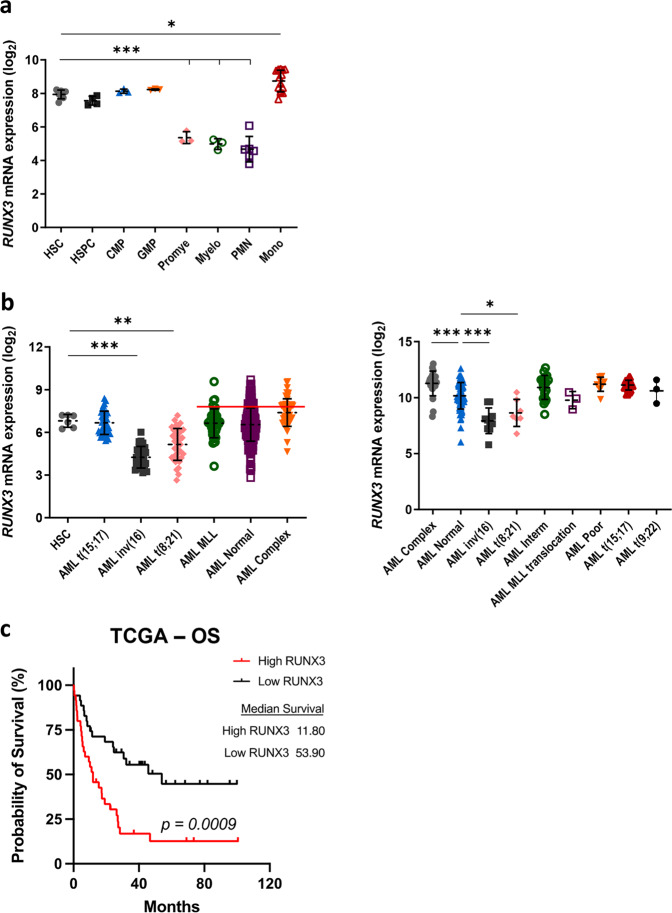
*RUNX3* mRNA expression in normal myeloid cell subpopulations and AML. **a** *RUNX3* mRNA expression in distinct human bone marrow (BM) hematopoietic cell subsets based on cell surface marker expression. HSC Hematopoietic stem cell, HSPC Hematopoietic stem and progenitor cell, CMP Common myeloid progenitor, GMP Granulocyte-monocyte progenitor, Promye - Promyelocyte, Myelo - Myelocyte, PMN - Polymorphonuclear cell, Mono - CD14^+^ monocyte. Human HSC are from GSE17054 (ref. [16]); Human GMP are from GSE19599 (ref. [17]); Human monocytes are from GSE11864 (ref. [18]) and E-MEXP-1242 (ref. [23]). 204197_s_at probeset used. Data indicate mean ± 1 SD (*n* ≥ 3). Statistical analysis was performed using ANOVA with Tukey’s multiple comparisons test, *p < *0.05*; ***p < *0.001 vs* HSC BM. **b** *(Left panel) RUNX3* mRNA expression in healthy BM cells and in distinct leukemic subtypes based on cytogenetics of patients. HSC data obtained from GSE42519 (refs. [19, 20]), and AML data obtained from GSE13159 (refs. [21, 22]) using 204197_s_at probeset. Data indicate mean ± 1 SD (n ≥ 6). Red line denotes >mean + 2 SD of HSC *RUNX3* expression, above which *RUNX3* is considered overexpressed in AML patients. Statistical analysis was performed using ANOVA with Tukey’s multiple comparisons test, **p < *0.01*; ***p < *0.001* vs HSC. (*Right panel*) *RUNX3* mRNA expression in different AML subtypes. RNA-seq data obtained from TCGA [24] using cBioPortal [26, 27]. AML Complex – AML with complex cytogenetics; AML Normal – AML with normal karyotype; AML Interm – AML with intermediate cytogenetic risk; AML Poor – AML with poor cytogenetic risk. RNA-seq data obtained from TCGA [24]. Data indicate mean ± 1 SD (*n* ≥ 3). Statistical analysis was performed using ANOVA with Tukey’s multiple comparisons test, *p < *0.05*; ***p < *0.001* vs AML Normal. **c** Kaplan-Meier overall survival curve for AML patients stratified according to upper and lower *RUNX3* mRNA expression quartiles. Data obtained from TCGA [24] using cBioPortal [26, 27]. RUNX3 upper quartile (*n* = 35); RUNX3 lower quartile (*n* = 35). Untreated and t(15;17) AML patients were excluded from this analysis. Survival analysis was performed using the Long-Rank test between high and low *RUNX3* expression groups.

To determine the pattern of *RUNX3* expression in normal human myeloid development, gene expression profiling was performed. Microarray-based transcriptomic data shows that *RUNX3* mRNA expression decreases significantly during granulopoiesis but is at higher levels in monocytes (Fig. 1a). Similar findings were observed in an additional transcriptomic dataset (Supplementary Fig. S2a). Overall, these data demonstrate that *RUNX3* is differently expressed during normal myeloid development and suggest different roles for RUNX3 during monocytic and granulocytic differentiation.

### RUNX3 is variably expressed across AML subtypes and its expression is associated with poorer overall survival of patients

To examine *RUNX3* mRNA expression levels in different AML subtypes, publicly available transcriptomic datasets were analyzed. As previously reported [4], significant downregulation of *RUNX3* levels was observed in inv(16) and t(8;21) AML compared to normal hematopoietic stem cells (HSC; Fig. 1b). Conversely, a significant proportion of non-CBF AML had elevated or reduced levels of *RUNX3* mRNA compared to normal HSC (Fig. 1b left panel). For instance, 26% of non-CBF AML patients overexpressed *RUNX3* in comparison with HSC whilst 13% had lower levels, similar to that observed in CBF leukemia. *RUNX3* overexpression in the former group was up to 6.5 times that seen in HSC. Further, patients with higher *RUNX3* expression had lower OS and disease-free survival (DFS) compared to low expressing *RUNX3* patients (Fig. 1c and Supplementary Fig. S2b respectively). OS analysis of non-CBF AML patients shows that RUNX3 overexpression is associated with poor survival (Supplementary Fig. S2c left panel). An additional AML dataset (including all patients) shows a similar observation (Supplementary Fig. S2c right panel). In terms of clinical attributes, high *RUNX3* patients are significantly associated with poor cytogenetic risk AML subtypes, low white blood cell counts, and later diagnosis age compared to low *RUNX3* patients (Supplementary Fig. S3). Overall, these data show that downregulation of *RUNX3* is associated with good prognosis AML subtypes, whereas increased *RUNX3* levels relate to worse prognosis of AML patients.

### Overexpression of RUNX3 inhibits human myeloid development

Considering that RUNX3 is frequently overexpressed in AML, we next examined the impact of RUNX3 overexpression on myeloid development by transducing normal human CD34^+^ HSPC with recombinant retrovirus co-expressing RUNX3 and DsRed. Cellular growth and differentiation of transduced HSPC were followed over 13 days. Overexpression of RUNX3 at protein and mRNA levels was successfully confirmed in RUNX3 transduced cells compared with controls (Supplementary Fig. S4).

**Fig. 2:**
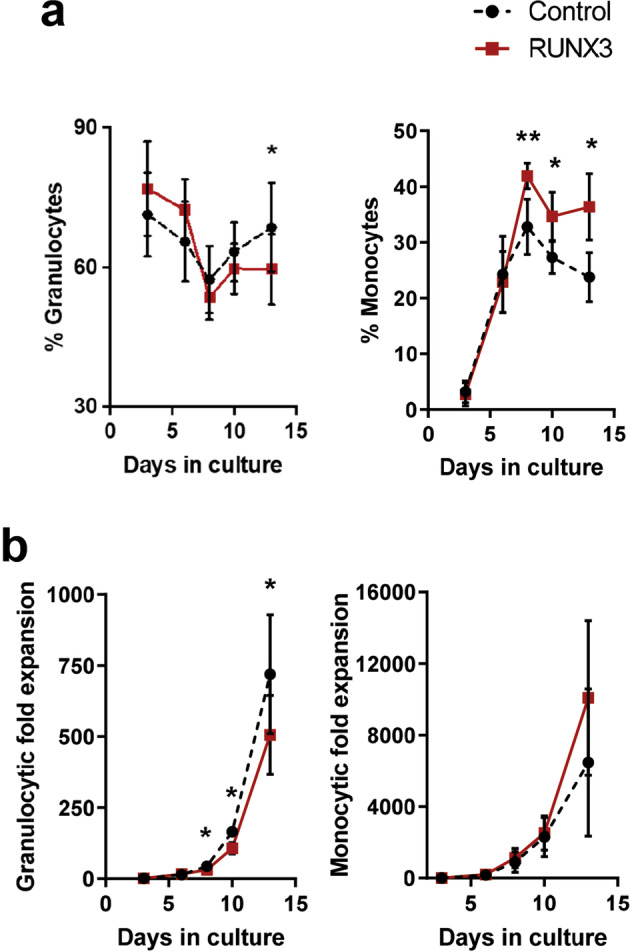
Overexpression of RUNX3 inhibits granulocytic growth and disrupts the balance between monocytic and granulocytic populations during myeloid development. **a** Summary data of the percentage of granulocytic committed cells (*Left panel*, CD13^–/+^CD36^–^), and monocytic committed cells (*Right panel*, CD13^+^CD36^+^) in both control and RUNX3 cultures. Data indicate mean ± 1 SD (*n* ≥ 3). Significant difference of RUNX3-expressing cells from controls was analyzed by paired t-test, *p < *0.05*; **p < *0.01*. **b** Cumulative fold expansion of control and RUNX3 myeloid cultures in terms of granulocytic committed cells (*Left panel*), and monocytic committed cells (*Right panel*) cultured over 13 days in culture medium containing IL-3, SCF, G-CSF and GM-CSF. Data indicate mean ± 1 SD (*n* ≥ 3). Significant difference of RUNX3-expressing cells from controls was analyzed by paired *t*-test, *p < *0.05*.

To assess the growth of cells committed to different lineages, CD13 and CD36 cell surface markers were used to discriminate between erythroid, monocytic and granulocytic populations (Supplementary Fig. S1). We found that RUNX3 overexpression progressively reduced the proportion of granulocytic cells in favor of monocytes (Fig. 2a). These observations are supported by the slower growth of granulocytic cells overexpressing RUNX3 whilst monocytic growth was not significantly affected (Fig. 2b). Taken together, these data show that RUNX3 overexpression selectively suppresses the growth of cells committed to granulocytic lineage.

**Fig. 3:**
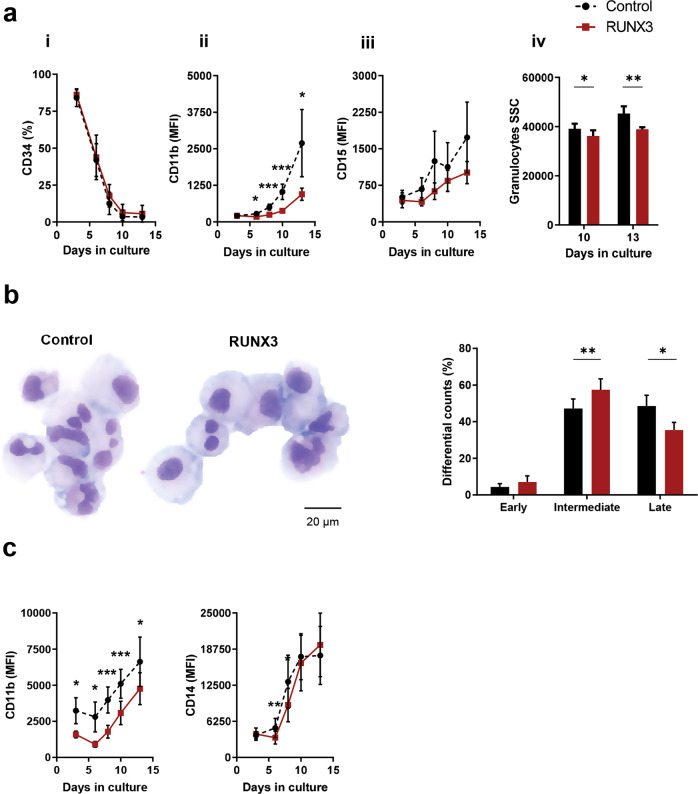
Overexpression of RUNX3 inhibits granulocytic development of human HSPC. **a** Summary data of granulocytic development of control and RUNX3 cultures. (**i**) CD34 percentage, (**ii**) CD11b expression, (**iii**) CD15 expression, and (**iv**) side scatter (SSC) gated on CD13^–/+^CD36^–^ granulocytic committed cells grown over 13 days in culture medium containing IL-3, SCF, G-CSF and GM-CSF. Data indicate mean ± 1 SD (*n* ≥ 3). Significant difference of RUNX3-expressing cells from controls was analyzed by paired t-test, *p < *0.05*; **p < *0.01*; ***p < *0.001*. **b** (*Left panel*) Control and RUNX3 cells analyzed on day 17 of differentiation with May-Grünwald-Giemsa staining. (*Right panel*) Differential morphology counts categorized into early (myeloblasts/promyelocytes), intermediate (myelocytes/metamyelocytes) and late phase (band/segmented granulocytic cells). Only cells from granulocytic lineage were scored for this analysis. Data indicate mean ± 1 SD (*n* ≥ 3). Significant difference of RUNX3-expressing cells from controls was analyzed by paired t-test, *p < *0.05*; **p < *0.01*. **c** Summary data of monocytic development of control and RUNX3 cultures according to *(Left panel)* CD11b expression, and *(Right panel****)*** CD14 expression gated on CD13^+^CD36^+^ monocytic committed cells. Data indicate mean ± 1 SD (*n* ≥ 3). Significant difference of RUNX3-expressing cells from controls was analyzed by paired t-test, *p < *0.05*; **p < *0.01*; ***p < *0.001*.

To determine the impact of RUNX3 overexpression on myeloid differentiation, expression of cell surface markers was analyzed over time by flow cytometry. Normal myeloid differentiation is characterized by the rapid loss of CD34 and upregulation of CD11b [29, 30]. Further, granulocytic cells upregulate CD15 whereas monocytic cells upregulate CD14 expression [29, 30]. Although CD34 expression remained unaltered (Fig. 3a(i)), RUNX3 overexpression suppressed the normal differentiation of granulocytes in culture evidenced by the downregulation of CD11b expression (2.8-fold), CD15 expression (1.8-fold), and a significant reduction in granularity (SSC; 1.2-fold) on day 13 of culture (Fig. 3a(ii–iv)). Morphological assessment showed that RUNX3 overexpression retained cells in a more intermediate stage of myeloid differentiation, with a significant 1.4-fold reduction in the number of band/segmented mature cells compared to controls (late phase of granulocytic development) (Fig. 3b). Overall, these data suggest that granulocytic cells overexpressing RUNX3 were more immature than control cells.

**Fig. 4:**
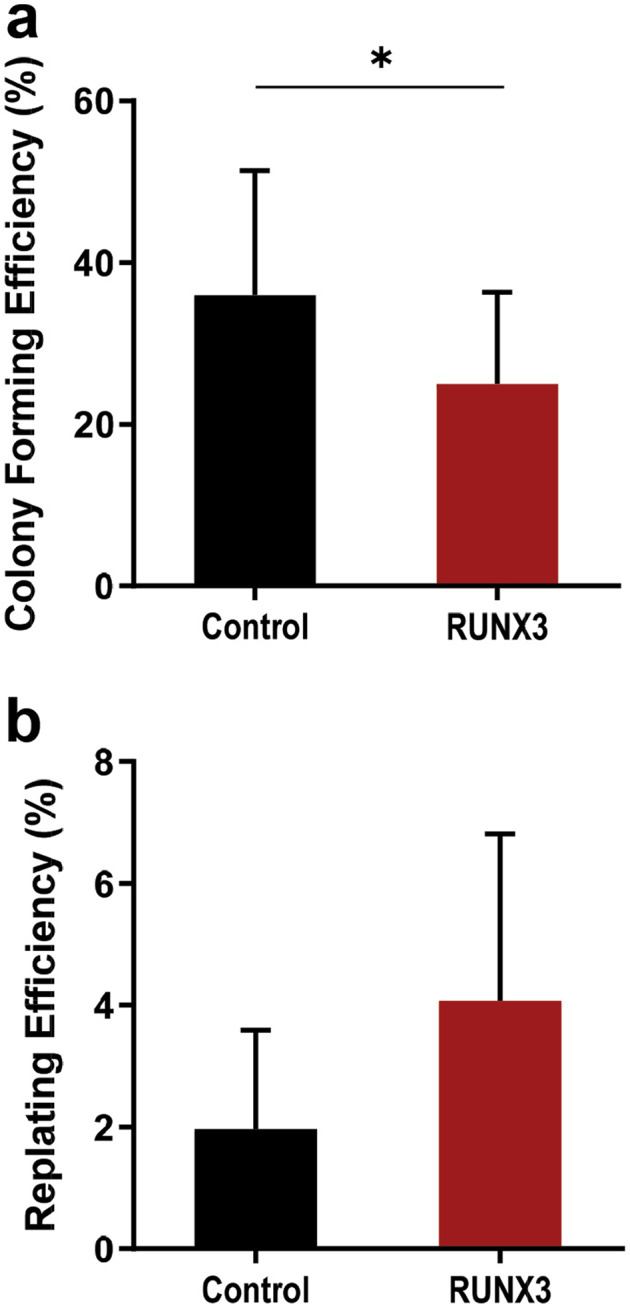
RUNX3 overexpression inhibits myeloid colony formation of human HSPC. **a** Summary data of myeloid colony forming efficiency for control and RUNX3 cultures following 7 days of growth in liquid culture containing IL-3, SCF, G-CSF and GM-CSF. Transduced cells were sorted for DsRed positivity on day 3 by FACS. Clusters were not included in this analysis. Data indicate mean ± 1 SD (*n* = 6). Significant difference of RUNX3-expressing cells from controls was analyzed by paired t-test, *p < *0.05*. **b** Self-renewal potential assessed by a single replating round of control and RUNX3 cultures in the same conditions as previously. Clusters were not included in this analysis. Data indicate mean ± 1 SD (*n* = 3).

Interestingly, RUNX3 overexpression in monocytic cells also inhibited CD11b expression (2.3-fold on day 8), and delayed CD14 upregulation (1.5-fold on day 6, Fig. 3c) compared to controls, suggesting that monocyte development may also be impacted (though to a lesser extent). Under clonal conditions, RUNX3 overexpression inhibited myeloid colony formation by 1.5-fold compared with control cells (Fig. 4a). In contrast, a serial replating assay showed that RUNX3 cells were able to form more colonies than controls, though not statistically significant (Fig. 4b), suggesting that RUNX3 may promote the self-renewal potential of these cells. Overall, these results suggest that RUNX3 expression suppresses the colony forming ability of myeloid progenitors as well as terminal differentiation of granulocytic cells with possible impact on monocytic lineage development.

We next studied whether RUNX3 knockdown (KD) affected myeloid cell development using 3 different shRNA constructs. As previously reported, *RUNX3* expression was successfully reduced by approximately 50% in sorted HSPC (day 3) [12]. RUNX3 KD did not impact the myeloid growth and development of human HSPC (Supplementary Fig. S5). Likewise, there was little impact of RUNX3 KD on AML cell line growth or apoptosis (Supplementary Figs. S6 and S7). However, we cannot conclude that RUNX3 expression is redundant given the incomplete KD of RUNX3.

### RUNX3 overexpression fails to rescue the phenotype of RUNX1::ETO-expressing HSPC

In contrast to non-CBF leukemias, downregulation of *RUNX3* expression has been observed in t(8;21) AML patients (Fig. 1b and [4]) and human HSPC (Supplementary Fig. S8). To determine whether the repression of RUNX3 seen in t(8;21) is important for the RUNX1::ETO phenotype, RUNX3 was overexpressed in RUNX1::ETO-expressing HSPC (Supplementary Fig. S9). Cells expressing RUNX1::ETO were used as control in these experiments as the effect on differentiation has been previously established [30]. In bulk liquid culture, increased RUNX3 expression in RUNX1::ETO-expressing cells had no significant effect on cell proliferation compared to cells expressing RUNX1::ETO alone (Supplementary Fig. S10a(i) and b(i)). Further, no significant differences were observed in the proportions of cells in granulocytic and monocytic populations (Supplementary Fig. S10a(ii, iii) and b(ii, iii)).

**Fig. 5:**
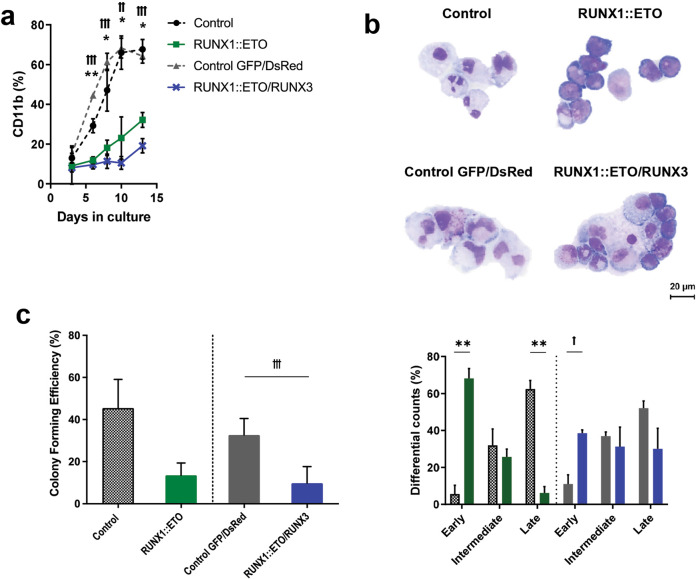
Expression of RUNX1::ETO in combination with RUNX3 does not rescue the myeloid defects induced by RUNX1::ETO alone in human HSPC. **a** Summary data of CD11b expression in terms of percentage in granulocytic cells over time for GFP^+^ control and RUNX1::ETO cultures, and GFP^+^DsRed^+^ control and RUNX1::ETO/RUNX3 cells. Data indicate mean ± 1 SD (*n* = 3). Significant difference of RUNX1::ETO cells vs control was analyzed by paired t-test, *p < *0.05*; **p < *0.01*. Significant difference of RUNX1::ETO/RUNX3 cells *vs* GFP^+^DsRed^+^ controls was analyzed by paired t-test, ^††^*p* < *0.01*, ^†††^*p* < *0.001*. **b** (*Top panel*) Single and double transduced cells analyzed on day 17 of differentiation with May-Grünwald-Giemsa staining. (*Lower panel*) Differential counts for all cultures with morphology categorized into early (myeloblasts/promyelocytes), intermediate (myelocytes/metamyelocytes) and late phase (band/segmented granulocytic cells). Data indicate mean ± 1 SD (*n* ≥ 3). Significant difference of RUNX1::ETO cells vs control was analyzed by paired t-test, **p < *0.01*. Significant difference of RUNX1::ETO/RUNX3 cells *vs* GFP^+^DsRed^+^ controls was analyzed by paired t-test, ^†^*p* < *0.05*. **c** Myeloid colony forming efficiency of single transduced control and RUNX1::ETO cells and double transduced control GFP/DsRed and RUNX1::ETO/RUNX3 cells following 7 days of growth in liquid culture containing IL-3, SCF, G-CSF and GM-CSF. Single transduced cultures were sorted for GFP positivity on day 3 by FACS, whereas double transduced cells were sorted for GFP and DsRed positivity in the same conditions as previous. Data indicate mean ± 1 SD (*n* = 3). Significant difference of RUNX1::ETO/RUNX3 cells *vs* GFP^+^DsRed^+^ controls was analyzed by paired t-test, ^†††^*p* < *0.001*.

Developmentally, both RUNX1::ETO and RUNX3-expressing cells impacted granulocytic differentiation characterized by a suppression of CD11b expression compared to controls (3.4-fold vs controls, Fig. 5a) and immature morphology (Fig. 5b). However, concomitant expression of RUNX3 had no significant impact on the phenotype conferred by RUNX1::ETO alone (Supplementary Fig. S11).

Under clonal conditions, simultaneous expression of RUNX1::ETO and RUNX3 maintained the suppression of colony formation of myeloid and erythroid progenitors induced by RUNX1::ETO alone (Fig. 5c, Supplementary Fig. S12). Taken together, these data suggest that RUNX3 overexpression does not relieve the differentiation impairment caused by RUNX1::ETO in human HSPC nor the deleterious effects of RUNX1::ETO on myeloid colony formation. Therefore, the hypothesis that RUNX3 downregulation by RUNX1::ETO plays a causal role in the pathogenesis of t(8;21) AML disease is not supported by these data.

### RUNX3 overexpression favors transcriptional repression and disrupts cell communication and immunity-related processes in human HSPC

**Fig. 6:**
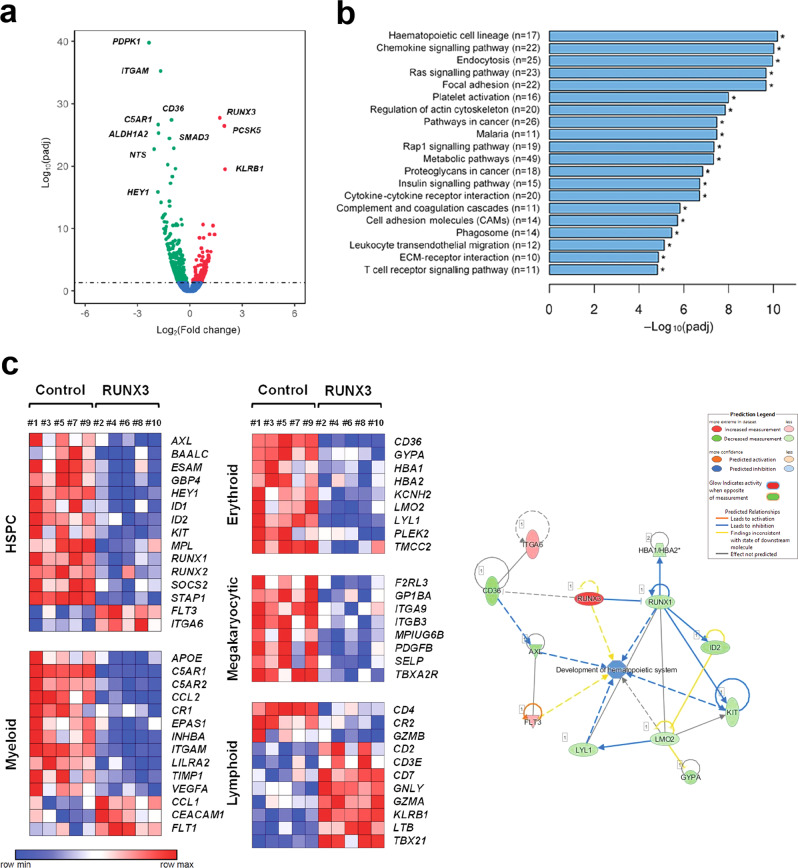
RUNX3 overexpression induces transcriptional dysregulation in human HSPC. **a** Volcano plot identifying DE genes between control and RUNX3 HSPC. Statistically significantly up- and downregulated genes are highlighted in red and green, respectively. **b** Bar plot showing the enrichment scores in terms of negative logarithm of the adjusted p-value (padj) of enriched terms using KEGG Pathway Analysis (www.genome.jp/kegg/pathway.html). KEGG terms with padj <0.05 are significantly enriched. n indicates the number of DE genes included in each KEGG pathway. **c** (*Left panel*) Heat maps representing the changes in expression levels of genes related to HSPC, myeloid, erythroid, megakaryocytic, and lymphoid compartments in control and RUNX3 human HSPC. BioProfiler in IPA^®^ was used to identify genes associated with human hematopoiesis. Data indicate normalized trimmed mean of M (TMM) expression values; box color is determined by low (blue) or high (red) gene expression levels. Each column represents an independent experiment (*n* = 5). (*Right panel*) Network with predicted interactions between key genes dysregulated by RUNX3 overexpression that are involved in hematopoietic differentiation of human HSPC. Most interactions between this set of dysregulated genes have been reported in the literature, denoted by blue lines (repression) or orange (transactivation), arrow heads indicate direction and effect. Network generated using IPA^®^ software and the Molecular Activity Predictor algorithm.

The above data suggest that RUNX3 overexpression impairs granulocytic cell development. To determine the impact of RUNX3 overexpression on the transcription of developmental drivers of hematopoiesis, RNA-seq was performed in RUNX3-expressing HSPC (Supplementary Fig. S13). Following infection, transduced cultures were >98% enriched for GFP expression by FACS (Supplementary Fig. S14) prior to RNA extraction. RUNX3 overexpression induced transcriptome changes in HSPC, characterized by 607 DE genes compared to control and was biased towards repression of genes (25% upregulated *vs* 75% downregulated; Fig. 6a). As expected, *RUNX3* was one of the most upregulated genes in the dataset (Supplementary Fig. S15). Enrichment analysis identified hematopoiesis as the main biological process dysregulated by RUNX3 overexpression in HSPC (Fig. 6b), supporting the above phenotypic data. Additional cellular processes were dysregulated, including the chemokine signaling pathway associated with cell migration, and the Ras signaling pathway involved in cell growth and survival. Further functional assays using the same experimental model showed that RUNX3 overexpression promoted the migration of HSPC towards an SDF-1 gradient, though not significant (Supplementary Fig. S16), supporting a possible leukemogenic role.

Dissection of key hematopoietic genes dysregulated by RUNX3 overexpression showed a general downregulation of HSC, myeloid, erythroid, and megakaryocytic genes, including the downregulation of developmental drivers and cell surface markers (Fig. 6c and Supplementary Fig. S17). RUNX3 overexpression was shown to induce significant changes in the transcription signatures related to HSPC function and maintenance including *KIT*, *LMO2*, *LYL1*, *HEY1*, *AXL* and *GPA*. Of note, transcriptional dysregulation of cell surface markers is limited given that the analysis was focused on immature cells and changes in hematopoietic drivers. In contrast, several lymphoid genes were upregulated by RUNX3 expression, possibly reflecting the important role for RUNX3 in lymphoid development [31]. A network of important dysregulated genes was generated using IPA^®^, highlighting the important relationships that ultimately result in the repression of hematopoietic development (Fig. 6c).

**Fig. 7:**
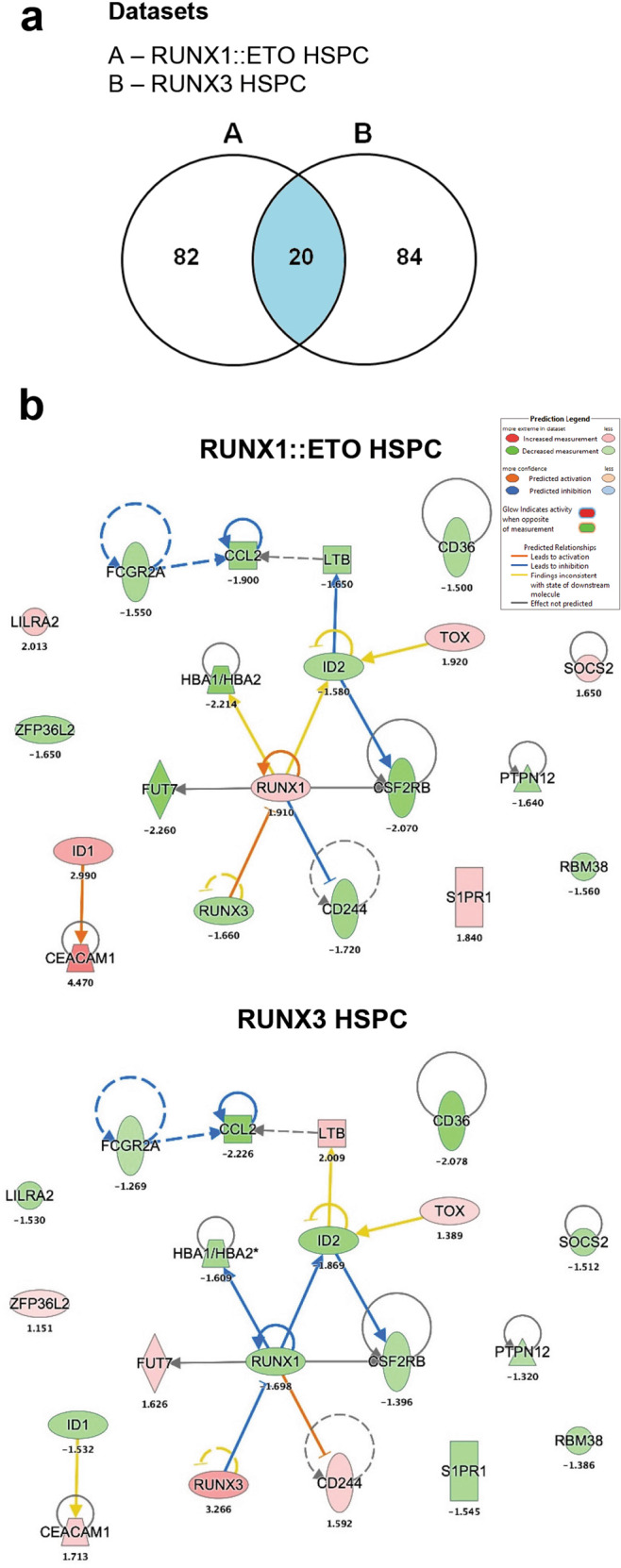
Differentially expressed genes comparison analysis of increased RUNX3 and RUNX1::ETO expression in human HSPC. **a** Venn diagram representing DE genes between increased RUNX3 and RUNX1::ETO expression HSPC datasets (GSE181059 and E-MEXP-583). For each dataset, DE genes were first filtered for hematopoietic relevant genes followed by a comparison analysis to identify common genes between datasets. **b** Networks of common hematopoietic-related genes (*n* = 20) with fold changes (green – downregulation, red – upregulation) and predicted interactions (blue line – repression, orange line – transactivation, arrowhead – direction and effect) for RUNX1::ETO and RUNX3 HSPC datasets. Networks generated using IPA^®^ software and the Molecular Activity Predictor algorithm.

Comparison with RUNX1::ETO-induced transcriptional dysregulation in human HSPC identified commonly dysregulated genes that have an important role in hematopoiesis (Fig. 7), suggesting that RUNX3 overexpression might be associated to leukemogenic events. *RUNX1* expression was upregulated in RUNX1::ETO HSPC dataset due to the ectopic expression of RUNX1::ETO in these cells. Even though *RUNX1* expression was opposing between datasets, expression of either RUNX3 or RUNX1::ETO as single abnormalities induced similar transcriptional effects on their common targets. This is explained by the negative dominant effect of RUNX1::ETO over RUNX1 native function, whereas RUNX3 directly repressed *RUNX1* expression by cross-regulation mechanisms. Furthermore, both RUNX3 and RUNX1::ETO expression in HSPC induced a similar downregulation of the transcriptional regulator *ID2*, which is also downregulated in CBF AML patients [32]. ID2 expression has been previously shown to be downregulated in translocations of the mixed lineage leukemia gene subtype of AML [32]. On the other hand, expression of transcriptional regulators *ID1* and *ZFP36L2* was opposing between both datasets. ID1 has been previously associated with t(8;21) AML initiation and progression, while the mRNA-destabilizing protein ZFP36L2 has been identified as a critical regulator of AML and potential therapeutic target [33]. Overall, RUNX3 overexpression was shown to induce significant transcriptional dysregulation in human HSPC, skewing transcriptional programming towards the lymphoid lineage.

## Discussion

RUNX3 has been shown to play a key role in hematopoiesis using different non-human cell models [34–37]. However, its role in human hematopoiesis and leukemia remains poorly understood. This study investigated RUNX3 expression during normal and malignant human hematopoiesis and the transcriptional dysregulation associated with abnormal RUNX3 levels in HSPC. RUNX3 overexpression was shown to inhibit human myelopoiesis and induce significant transcriptional repression of myeloid genes.

Although not as well studied as its family member RUNX1 in the context of hematopoiesis, RUNX3 is highly expressed in the hematopoietic system and is an important regulator of T cell differentiation [34, 38]. In normal myeloid development, *RUNX3* was generally expressed in cells with a decline during granulopoiesis. The fact that granulocytes were the most affected cells by RUNX3 overexpression (see below) suggests that RUNX3 downregulation is an important feature of granulocyte development. In AML, *RUNX3* mRNA overexpression is associated with poorer OS and DSF of patients. However, this could be accounted for by the negative association of *RUNX3* mRNA expression with CBF AML patients which have better outcomes [4]. Nonetheless, survival analysis of non-CBF AML patients suggests that high *RUNX3* levels remain associated with poor outcome. These findings support a recent study that identified *RUNX3* as one of three super-enhancer associated genes that is abnormally overexpressed in AML cells compared to normal hematopoietic cells, such as neutrophils, monocytes, and HSPC [39]. In childhood AML, increased *RUNX3* expression was associated with a shortened EFS and OS among patients [4, 40]. Recently, high *RUNX3* expression was shown to be associated with poorer survival of patients with myelodysplastic syndrome (MDS) [41]. Therefore, overexpression of RUNX3 could be associated with driving myeloid transformation.

Considering that RUNX3 is overexpressed in several AML subtypes except for CBF AML, the effects of RUNX3 overexpression on myeloid development were modeled using human HSPC. In bulk liquid culture, RUNX3-expressing cells grew slower than controls characterized by a suppression of granulocytic growth. RUNX3-mediated growth suppression in myeloid cells was accompanied by a lineage imbalance, and an inhibition of myeloid differentiation as CD11b expression was strongly downregulated by RUNX3. The close relationship between RUNX3 and CD11b expression has been previously described in dendritic cells using RUNX3-deficient mouse models [42]. RUNX3 was shown to occupy the genomic loci of *Itgam*, with its loss leading to increased expression of CD11b in mouse dendritic cells. Under clonal conditions, RUNX3 overexpression negatively affected the colony formation capacity of myeloid cells. As well as its impact on myeloid development, we have also shown that RUNX3 overexpression inhibits erythroid differentiation [12]. Since inhibition of myeloid differentiation is a defining feature of AML, these data suggest a role for RUNX3 overexpression in the pathogenesis of this disease.

Given the downregulation of *RUNX3* expression in CBF AML patients, and to fully assess its importance in normal human myeloid development, the effects of RUNX3 KD in cell growth and differentiation were further determined. In bulk liquid culture, KD of RUNX3 induced a modest reduction in the myeloid growth of cells, with no significant growth inhibition observed for RUNX3 KD monocytic and granulocytic cells. Recently, RUNX3 KD in human HSPC was shown to have little effect on myeloid development, with RUNX3 KD hematopoietic progenitors preserving their normal growth and granulocytic differentiation [43]. Particularly, RUNX3 downregulation in human HSPC did not affect myeloid colony forming capacity and caused minimal changes in granulocytic differentiation assessed by CD15 expression following 8 days in bulk liquid culture. These results are consistent with our current study. Considering that RUNX3 KD cells were able to differentiate into myeloid cells while aberrantly preserving myeloid markers such as CD123 and CD45RA, this study further suggests an additional role for RUNX3 in lineage resolution. Loss of RUNX3 was shown to cause a mild expansion of HSC and myeloid cells in aged mice [35], whereas disruption of both RUNX1 and RUNX3 in mice led to BM failure and myeloproliferative disease characterized by DNA repair defects [36]. To further explore the role of RUNX3 in AML, its expression was reduced in AML cell lines. RUNX3 KD did not impact on cell growth. This is in contrast to a recent study that showed that RUNX3 KD inhibits AML progression in MLL::AF9 mice by inducing DNA damage and apoptosis [39]. Overall, RUNX3 KD did not affect the normal program of myeloid differentiation of human HSPC or AML cell growth, with the caveat that RUNX3 was not completely suppressed.

*RUNX3* mRNA was shown to be significantly downregulated in both t(8;21) and inv(16) AML patients compared to normal HSC. Both abnormalities involve the heterodimeric protein complex CBF and are considered to have a good prognosis compared to other leukemic subtypes. To establish whether the repression of RUNX3 observed in t(8;21) patients is important for the RUNX1::ETO phenotype we ectopically expressed RUNX3 in the context of RUNX1::ETO to see if this could rescue the RUNX1::ETO phenotype which is characterized by inhibition of granulocytic development and colony formation [30]. We observed no evidence of rescue of this phenotype through ectopic RUNX3 expression either in bulk culture or in colony assays, suggesting that downregulation of RUNX3 in t(8;21) patients by itself does not contribute to the pathogenesis of t(8;21) AML.

We also carried out comparative transcriptomic analysis between transcriptomic changes induced by RUNX3 and RUNX1::ETO expression as single abnormalities in HSPC. This study suggests that both RUNX1::ETO and RUNX3 target similar processes in human HSPC, as both result in the suppression of RUNX1-mediated transcription. This may explain the phenotypic similarities observed between RUNX3 and RUNX1::ETO overexpression as single abnormalities in human HSPC [30]. Interestingly, *ID2* downregulation was observed in both RUNX3 and RUNX1::ETO HSPC datasets. Low ID2 expression has been previously associated with poor prognosis in MLL and t(8;21) AML patients, and its overexpression inhibited MLL::AF9- and RUNX1::ETO9a-driven leukemia progression and maintenance in mice [32]. Furthermore, RNA-seq studies in a TET2-deficient MDS mouse model overexpressing RUNX3 showed a positive enrichment in the expression of RUNX1::ETO target genes [41]. This study suggests that RUNX3 overexpression in this context suppresses RUNX1 transcriptional function, which has been shown to be downregulated by RUNX1::ETO.

Overexpression of RUNX3 led to a significant dysregulation of HSPC transcriptome. Enrichment analysis showed a significant correlation between these changes and important biological processes, such as hematopoiesis and cell movement. *PDPK1* was the most downregulated gene in RUNX3-expressing HSPC. Its encoded protein, PDK1 is a master kinase essential for cell survival and development in many species [44]. Besides its important role in HSC survival, PDK1 has been implicated in hematopoietic development with its loss impairing erythroid and myeloid colony formation and the terminal differentiation of embryonic cells [45]. Uniquely, downregulation of *HEY1*, a downstream target of the canonical Notch signaling pathway which plays important roles in cellular growth, differentiation, and fate choices [46], was the only change in TFs present in the top 10 DE genes. Notch signaling has been shown to promote erythroid differentiation of human CD34^+^ cells [47], and a connection between this pathway and SCF signaling during erythropoiesis has been previously reported [48]. Expression of HEY1 has been demonstrated in HSC and committed myeloid progenitors, which suggests an important role in hematopoiesis [49, 50]. *Hey1* KO zebrafish showed significantly decreased mature erythroid cells and diminished expression of *runx1*, which suggests this TF is required for definitive hematopoiesis and plays a critical role in the emergence of HSC [50].

Additional RUNX family members, *RUNX1* and *RUNX2*, were also significantly downregulated by RUNX3 overexpression in HSPC. RUNX1 is required for HSC emergence [51, 52], as well as definitive hematopoiesis resulting in midgestational death of RUNX1-deficient mice [53, 54]. RUNX3 overexpression in TET2-deficient mice was shown to significantly inhibit RUNX1 expression, as well as its target genes *Cebpa* and *Csf1r* [41]. In addition, AML patient samples from the TCGA dataset show a significant negative correlation between *RUNX3* and *RUNX1* mRNA expression [24]. Considering this evidence, and the possible redundancy between both TFs previously described in the literature [36, 55, 56], RUNX3-induced suppression of erythroid and myeloid developmental might be in part due to the downregulation of RUNX1 and dysregulation of its target genes. Nevertheless, essential and non-redundant functions of RUNX1 and RUNX3 have been previously demonstrated [34].

RUNX3 overexpression was shown to upregulate the expression of *ITGA6* (CD49f) and *FLT3*, cell surface markers associated with different HSC populations. These findings together with the phenotypic and morphological effects of RUNX3 overexpression in HSPC (see above) support an inhibition of early myeloid development by RUNX3. Furthermore, RUNX3 overexpression upregulated the expression of *KLRB1*, *GNLY, TBX21, LTB* and *GZMA*, among other lymphoid-related genes. AML patient data from the TCGA dataset shows a significant positive correlation between RUNX3 and the expression of these genes [24]. For instance, *KLRB1* (CD161 receptor) is mainly expressed in lymphoid cells, particularly NK cells [57–59]. ChIP-seq analysis has shown that RUNX1 binds to *KLRB1* promoter in pre-B cells [60]. Previously, RUNX3 has been shown to strongly drive CD8^+^ T cell development while suppressing CD4 expression [38]. Therefore, induction of CD8^+^ T cell-related genes such as *GNLY*, *GZMA*, or *TBX21* could suggest lineage misprogramming by RUNX3. Further support for the promotion of lymphoid genes by RUNX3 comes from the downregulation of *ID2* expression in HSPC. Reduction of ID2 expression in cord blood HSC has been shown to increase lymphoid potential, whereas its overexpression biased myelo-erythroid differentiation [61]. Considering the role of RUNX3 in the lymphoid lineage [31], this study suggests the potential for RUNX3 overexpression to transcriptionally misprogram cells to a lymphoid fate.

In conclusion, this study showed that increased expression of RUNX3 in human HSPC inhibits normal human myeloid development and induces a significant transcriptional repression of genes. This study also provides evidence of overlap of RUNX3 function with t(8; 21) in the pathogenesis of AML.

## Supplementary information

Supplemental Material and Figures
