# Supplementary material for: Increased expression of RUNX3 inhibits normal human myeloid development
Source: Leukemia. 2022 Apr 30;36(7):1769–80. doi: 10.1038/s41375-022-01577-2 (PMC9252899; doi:10.1038/s41375-022-01577-2)
Supplement: Supplementary file 1 — Supplemental Material and Figures [file 41375_2022_1577_MOESM1_ESM.pdf]

Supplemental Material and Methods

Source and structure of plasmids

A retroviral vector co-expressing RUNX3 and DsRed was generated by directional cloning of *RUNX3* (NM_001031680.2) into *Bam*H1/*Eco*R1 sites of PINCO vector. The source of plasmids used for both RUNX3 overexpression and knockdown (KD) studies in hematopoietic stem progenitor cells (HSPC) are listed in Supplemental Table S1.

Supplemental Table S1. Summary of retro- and lentiviral systems used for overexpression and KD studies.

All vectors include the *ampicillin resistance* gene. Puro – Puromycin; NM Number – National Center for Biotechnology Information (NCBI) reference sequence (RefSeq) database transcript accession number; TRCN Number – The RNAi Consortium shRNA Clone ID number. PINCO was a kind gift from gift of Pier Pelicci, European Institute of Oncology, Milan, Italy [1]. RUNX3 shRNA KD vectors were purchased from VectorBuilder (Guangzhou, China).

Plasmid	Gene/Target sequence	Selectable marker
PINCO	Stuffer fragment	DsRed
PINCO	RUNX3 [NM_001031680.2]	DsRed
PINCO	Stuffer fragment	GFP
PINCO	RUNX1::ETO [2]	GFP
PINCO	RUNX3 [NM_001031680.2]	GFP
pLV	Scramble shRNA	GFP/Puro
pLV	RUNX3 shRNA 1 [TRCN0000235676]<br>5’ GTTCAACGACCTTCGCTTCGT 3’	GFP/Puro
pLV	RUNX3 shRNA 2 [TRCN0000235675]<br>5’ ACCACCTCTACTACGGGACAT 3’	GFP/Puro
pLV	RUNX3 shRNA 3 [TRCN0000235672]<br>5’ TGGCAGGCAATGACGAGAACT 3’	GFP/Puro

Isolation, infection, and culture of human HSPC

Human neonatal cord blood was obtained from healthy full-term pregnancies at the University Hospital Wales, Cardiff, UK. These were obtained with informed consent and with approval from the South East Wales Research Ethics Committee in accordance with the 1964 Declaration of Helsinki. Normal human HSPC were isolated, cultured and transduced with retro/lentivirus as previously described [2]. Briefly, HSPC were infected with retro- or lentivirus by centrifugation for 120 minutes at 2200 *x g* and room temperature (RT) in 24-well plates pre-coated with 30 µg/mL RetroNectin® (Takara, Paris, France) [3]. Following centrifugation, the retroviral supernatant was removed and HSPC were added to the wells (70-140 000 cells/well). The infection procedure was repeated on the following day to improve the transduction efficiency. Following infection (day 3 of culture), cells were maintained in Iscove's Modified Dulbecco's Medium (IMDM; Fisher Scientific, Loughborough, UK) supplemented with 5 ng/mL of IL-3, SCF, G-CSF, and GM-CSF (BioLegend, London, UK).

For overexpression experiments, three cultures were generated: mock (no infection), control (PINCO expressing DsRed alone) and RUNX3 co-expressing DsRed. For KD studies: mock, shRNA control GFP and 3 different shRNA targeting RUNX3 and co-expressing GFP were generated. To study the concomitant effects of RUNX3 and RUNX1::ETO expression in HSPC, four cultures were generated: control (PINCO expressing GFP alone) and RUNX1::ETO co-expressing GFP; control GFP/DsRed (HSPC transduced with both previous GFP and DsRed PINCO vectors) and RUNX3/RUNX1::ETO (HSPC transduced with both previous RUNX3 DsRed and RUNX1::ETO GFP PINCO vectors).

Phenotypic and differentiation analysis by flow cytometry

Transduced cultures were analyzed by flow cytometry at different time points using a panel of cell surface markers (Supplemental Table S2) as previously described [2]. CD13-APC in combination with CD36-biotin were used for lineage discrimination; Streptavidin PerCP-Cy5.5 was used as a second-step detection reagent. In addition, cells were incubated with one of the following Pacific Blue (PB)-labelled differentiation markers: CD11b, CD14, CD15 and CD34 (BioLegend, London, UK). All incubations were performed at 4°C, and reactions were controlled with the appropriate isotype-matched irrelevant antibody. Reagent concentrations were as recommended by the manufacturer.

Supplemental Table S2. Summary of antibodies used in flow cytometry.

NA – Not applicable; Cambridge Bio – Cambridge Biosciences; CST – Cell Signaling Technology; SCBT – Santa Cruz Biotechnology.

Antibody	Clone	Supplier
Anti-Human CD11b-Pacific Blue™ (PB)	ICRF44	BioLegend, London, UK
Anti-Human CD13-APC	WM15	BioLegend, London, UK
Anti-Human CD14-PB	HCD14	BioLegend, London, UK
Anti-Human CD15-PB	W6D3	BioLegend, London, UK
Anti-Human CD34-PB	581	BioLegend, London, UK
Anti-Human CD36-Biotin	NA	Cambridge Bio., Cambridge, UK
Mouse IgG1-PB	MOPC-21	BioLegend, London, UK
PerCP-Cy™5.5 Streptavidin	NA	BD Biosciences, Wokingham, UK

Morphology

Approximately 30 000 cells were centrifuged (using Cytospin 3, 60 *x g* for 5 minutes) in a pre-assembled cytospin sample chamber with glass slide. Slides were stained with May–Grünwald–Giemsa for morphology examination and scanned using Zeiss Axioscan Z1 slide scanner (Carl Zeiss, Cambridge, UK) at 20X magnification. Differential counts were performed using Zen Lite software (Carl Zeiss, Cambridge, UK) to determine the number of cells in separate developmental stages. Granulocytic cells in an early, intermediate, and late phase of development were defined as myeloblasts/promyelocytes, myelocytes/metamyelocytes and band/segmented cells, respectively. Monocytes were excluded from the differential counts.

Cell culture

AML cell lines used in this study included OCI-AML2, OCI-AML5 and TF-1, and were obtained from ATCC (LGC Standards, Middlesex, UK) and DSMZ (Braunschweig, Germany). All cells were cultured according to the supplier standard cell culture guidelines, under aseptic conditions and grown at 37°C in a 5% CO_2 humidified incubator. The genetic identity of the cell lines was confirmed by short tandem repeat (STR) at purchase and in 2021. Monthly monitoring for Mycoplasma contamination was performed and confirmed using the MycoAlert Detection Kit (Sigma).

Cell proliferation and apoptosis

Cultures were counted by flow cytometry using TO-PRO-3 (Fisher Scientific, Loughborough, UK), allowing the appropriate gating of viable cells. To determine the effects of increased and reduced levels of RUNX3 on AML cell growth, cells were plated

in 24-well culture dishes and their proliferation was followed over 3 to 8 days (depending on doubling time). Cell density was determined, and the cumulative fold expansion was plotted over time.

Changes in apoptotic cell death were determined using the Annexin V-APC Apoptosis Detection Kit (VWR International, Lutterworth, UK) following manufacturer's instructions. Briefly, $1x10^5$ cells were washed with PBS, followed by a second wash with 1X Binding buffer. Cells were stained with Annexin V-APC (BD Pharmingen™, Berkshire, UK) and incubated for 15 minutes at RT. Following washing, propidium iodide (Fisher Scientific, Loughborough, UK) was added to the cell suspensions. Samples were analyzed by flow cytometry within 4 hours.

Flow Cytometry

Flow cytometry data was acquired using a BD FACSCanto™II (BD Biosciences, Wokingham, UK). The threshold for GFP/DsRed positivity was determined using identically treated mock transduced cultures. At least 20 000 events were recorded for each sample at a medium flow rate. Data were analyzed using FCS Express v6 (De Novo Software, Pasadena, CA, USA). Debris was excluded from all analyses based on light scatter.

Western Blot

Cytosolic and nuclear proteins were extracted from $5x10^6$ HSPC using the Biovision Nuclear/Cytosol Fractionation Kit (Cambridge Bioscience, Cambridge, UK). Briefly, cells were pelleted and washed, followed by sequential incubations with extraction buffers. Cytosol fractions were separated, and nuclear proteins were

extracted using triethylammonium bicarbonate buffer (TEAB). Bradford protein assay was performed by measuring the absorbance of Bradford's reagent solution (Sigma Aldrich, Dorset, UK) at 595 nm.

SDS-PAGE was performed as previously described [4] using the NuPAGE® electrophoresis system (Fisher Scientific UK Ltd, Loughborough, UK). Detection of RUNX3 protein expression was determined using a primary rabbit monoclonal antibody (D6E2, Cell Signaling, London, UK) in conjunction with an anti-rabbit HRP Amersham ECL Advance Western Blotting Detection Kit (Cytiva, Little Chalfont, UK) according to the manufacturer's instruction. GAPDH (6C5, SCBT, Heidlberg, Germany) and Histone 1 (AE-4, Bio-Rad, Hertfordshire, UK) protein expression was assessed for equal loading purposes. Densitometry was performed using ImageJ v1.8 software (https://imagej.nih.gov/ij/) by plotting a histogram of peak intensity for each band. The peak area was used as an arbitrary intensity value to estimate the fold changes in protein expression. Data was corrected for loading (GAPDH/Histone 1 expression) and normalized against control cells.

Migration assay

The Transwell® cell migration assay measures the capacity of cell motility towards a chemoattractant gradient (e.g. stromal cell-derived factor 1, SDF-1) [5]. Initially, the transwell 24-well plate was pre-incubated with serum free growth medium or chemotaxis medium (IMDM containing 1% *v/v* BSA) by adding 600 μL medium to each lower chamber and 100 μL to the top of the filter membrane or transwell insert. In addition, serial dilutions of SDF-1 were prepared in chemotaxis medium at final concentrations of 0.36, 1.2, 3.6, and 12 μg/mL. Transduced HSPC (day 6) were counted and $1x10^5$ cells were washed and resuspended in 590 μL ($1.7x10^5$ cells/mL) of

chemotaxis medium. The medium in each transwell insert was replaced by 100 µL of cell suspension and 5 µL of SDF-1 at final concentrations of 3, 10, 30 and 100 ng/mL was added to the lower chambers. Cells were incubated at 37°C and 5% CO_2 for 4 hours, after which the number of migrated cells was counted by flow cytometry using 1 µg/mL 7-Aminoactinomycin D. A negative control to account for spontaneous migration was included by replacing the addition of SDF-1 for chemotaxis medium. The percentage of cell migration was calculated based on the number of cells present in the lower chamber in relation to the total number of cells measured in both compartments.

Transcriptome analysis

RNA concentration and purity were assessed using NanoDrop™ (Fisher Scientific UK Ltd, Loughborough, UK). *RUNX3* mRNA expression in human HSPC was validated by qRT-PCR using *GAPDH* as reference gene (Hs02786624_g1). Gene expression was assessed using QuantStudio™ 5 Real-Time PCR System (Fisher Scientific UK Ltd, Loughborough, UK). Gene expression data was analyzed using QuantStudio™ Design and Analysis software v1.5.1 by Thermo Fisher Scientific.

Sorted HSPC expressing RUNX3 (and respective control) were resuspended in Buffer RLT, and total RNA was extracted for RNA-seq purposes. Total RNA sample QC was performed using NanoDrop™ and Agilent 2100. Illumina NovaSeq 6000 was used to sequence the cDNA library with a read length of 150bp paired end, 20 million (M) reads per sample and a sequencing quality score for a given base (Q) ≥ 80%. Reads were mapped to the human reference genome (hg19) using HISAT2 algorithm. HTSeq package was used to determine gene expression levels using the union mode. Web-based tool Morpheus (www.software.broadinstitute.org/morpheus) was used to

generate expression heat maps of relevant differently expressed genes in control and RUNX3-expressing HSPC.

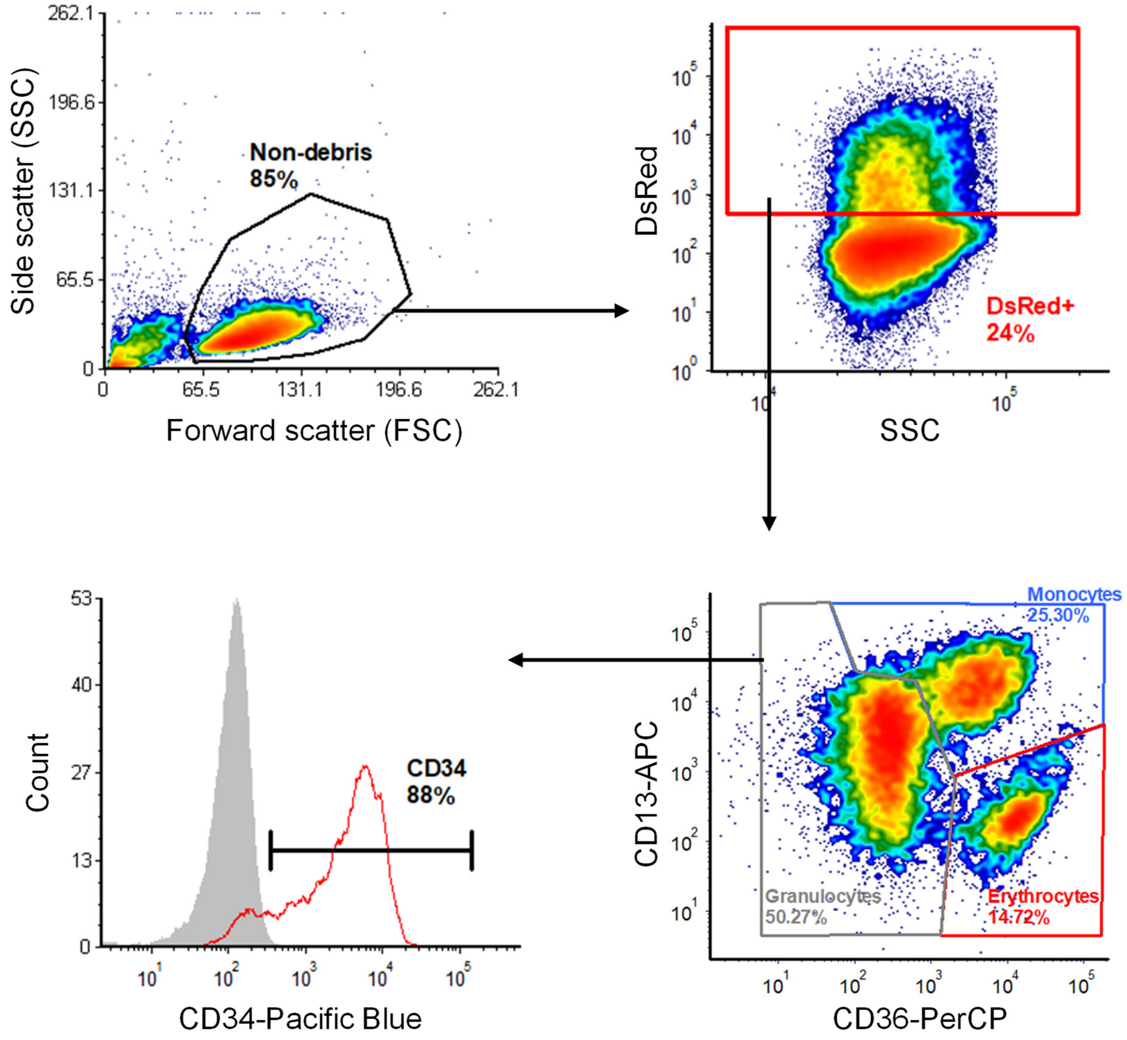

Supplemental Fig. S1. Gating strategy used for the analysis of myeloid growth and differentiation of HSPC by flow cytometry.

Representative flow cytometry histograms and bivariate density plots of transduced HSPC on day 6 of culture. Non-debris – gate used to exclude all debris from the analysis. $DsRed^{+}$ – gate used to analyze transduced HSPC. Granulocytes – gate used to analyze the growth and differentiation of granulocytic cells ($CD13^{-/+}CD36^{-}$); Monocytes – gate used to analyze the growth and differentiation of monocytic cells ($CD13^{+}CD36^{+}$); Erythrocytes – Gate used to assess the growth and percentage of erythroid-committed cells ($CD13^{-}CD36^{+}$) in culture. IgG-Pacific Blue stained HSPC – gray peak.

Supplemental Figures

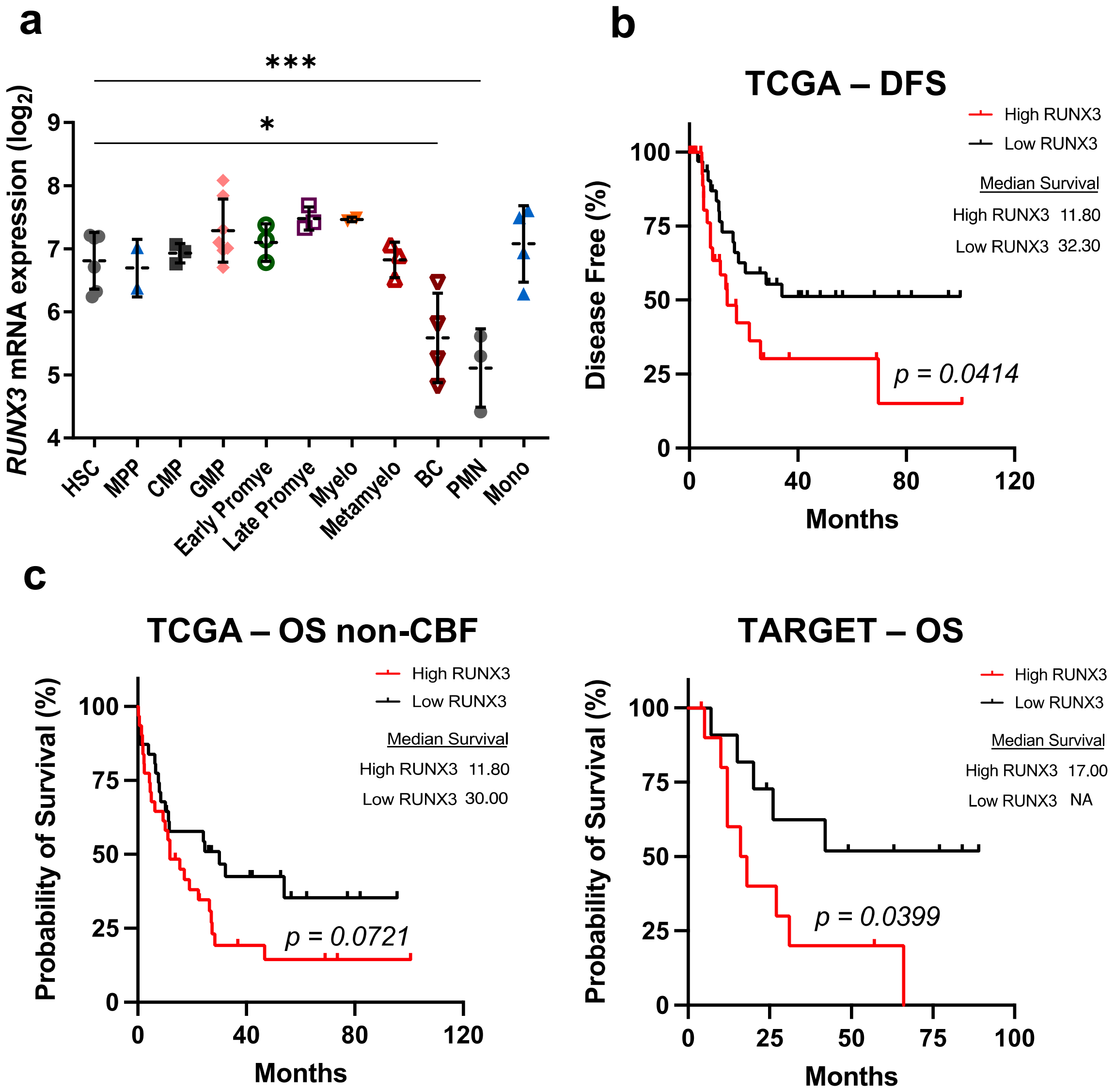

Supplemental Fig. S2. *RUNX3* mRNA expression in different hematopoietic cell subpopulations and AML.

a *RUNX3* mRNA expression in different human hematopoietic cell subtypes. MPP – multipotent progenitor cell; Metamyelo – metamyelocyte; BC – band cell-. Data obtained from GSE42519 (Ref. [6, 7]). Data indicate mean ± 1SD (n≥3). Statistical analysis was performed using ANOVA with Tukey's multiple comparisons test, *$p<0.05$*; ****$p<0.001$* vs HSC. **b** Kaplan-Meier disease free survival curve for AML patients stratified according to upper and lower *RUNX3* mRNA expression quartiles. Data obtained from TCGA [8] using cBioPortal [9, 10]. RUNX3 upper quartile (n=35); RUNX3 lower quartile (n=35). Untreated and t(15;17) AML patients were excluded from this analysis. Disease free survival analysis was performed using the Long-Rank test between high and low *RUNX3* expression groups. **c** *(Left panel)* Kaplan-Meier overall survival curve for non-CBF AML patients stratified according to upper and lower *RUNX3* mRNA expression quartiles. Data obtained from TCGA [8] using cBioPortal [9, 10]. RUNX3 upper quartile (n=31); RUNX3 lower quartile (n=31). CBF AML patients were additionally excluded from this analysis. Survival analysis was performed using the Long-Rank test between high and low *RUNX3* expression groups. *(Right panel)* Kaplan-Meier overall survival curve for AML patients stratified according to upper and lower *RUNX3* mRNA expression quartiles. Data obtained from the Therapeutically Applicable Research to Generate Effective Treatments (TARGET, https://ocg.cancer.gov/programs/target, phs000465) initiative, using cBioPortal [9, 10]. RUNX3 upper quartile (n=11); RUNX3 lower quartile (n=11). Survival analysis was performed using the Long-Rank test between high and low *RUNX3* expression groups. NA – Undefined.

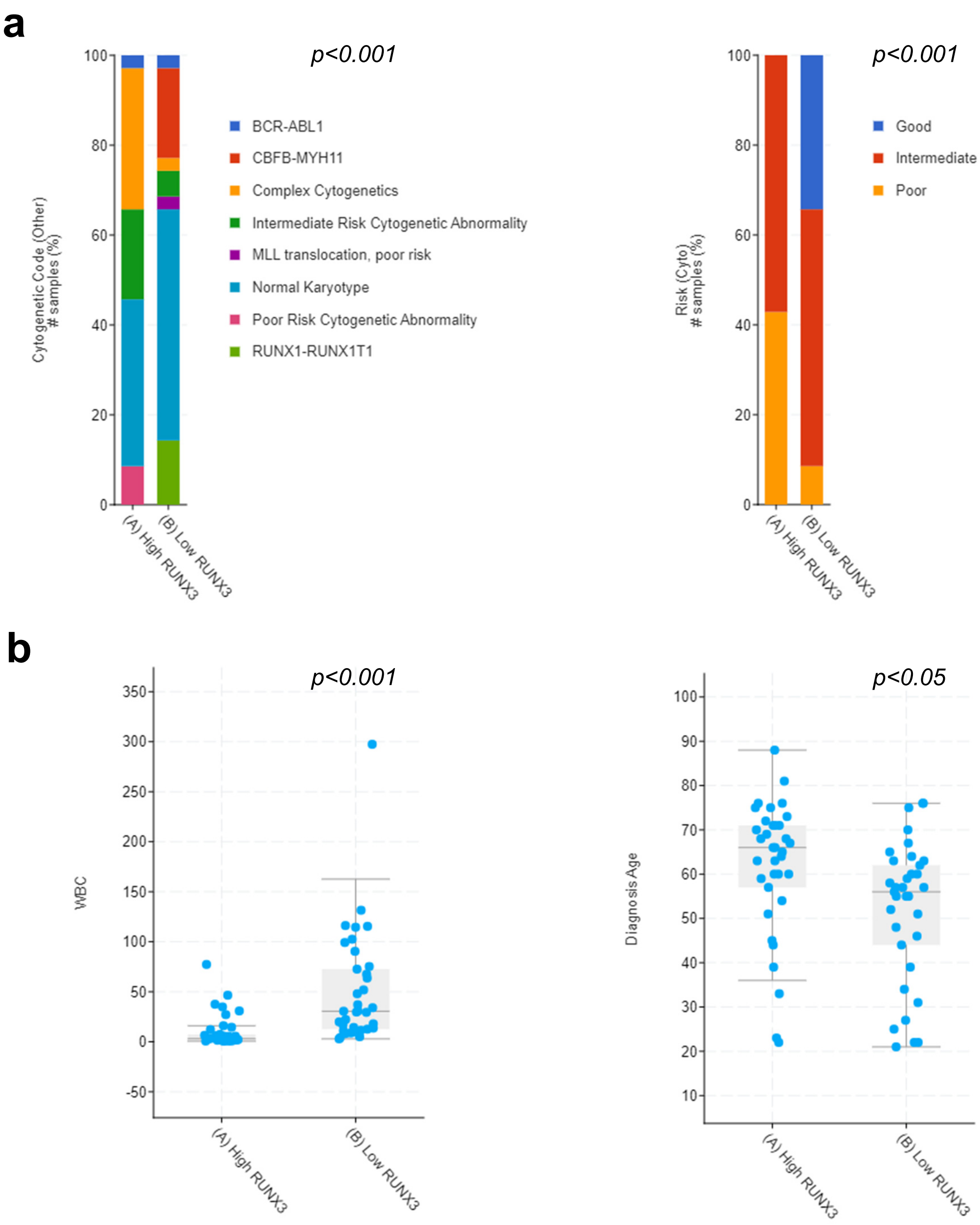

Supplemental Fig. S3. Increased *RUNX3* expression is associated with complex cytogenetics and poor prognosis in AML.

a *(Left panel)* Cytogenetic code of AML patients according to *RUNX3* expression. *(Right panel)* Cytogenetic risk of AML patients according to *RUNX3* expression. Data obtained from TCGA [8]. RUNX3 upper quartile n=35; RUNX3 lower quartile n=35. Statistical analysis performed using the Chi-squared test. **b** *(Left panel)* White blood cell (WBC) count of AML patients according to *RUNX3* expression. *(Right panel)* Diagnosis age of AML patients according to *RUNX3* expression. Data obtained from TCGA [8]. RUNX3 upper quartile n=35; RUNX3 lower quartile n=35. Statistical analysis performed using the Kruskal Wallis test.

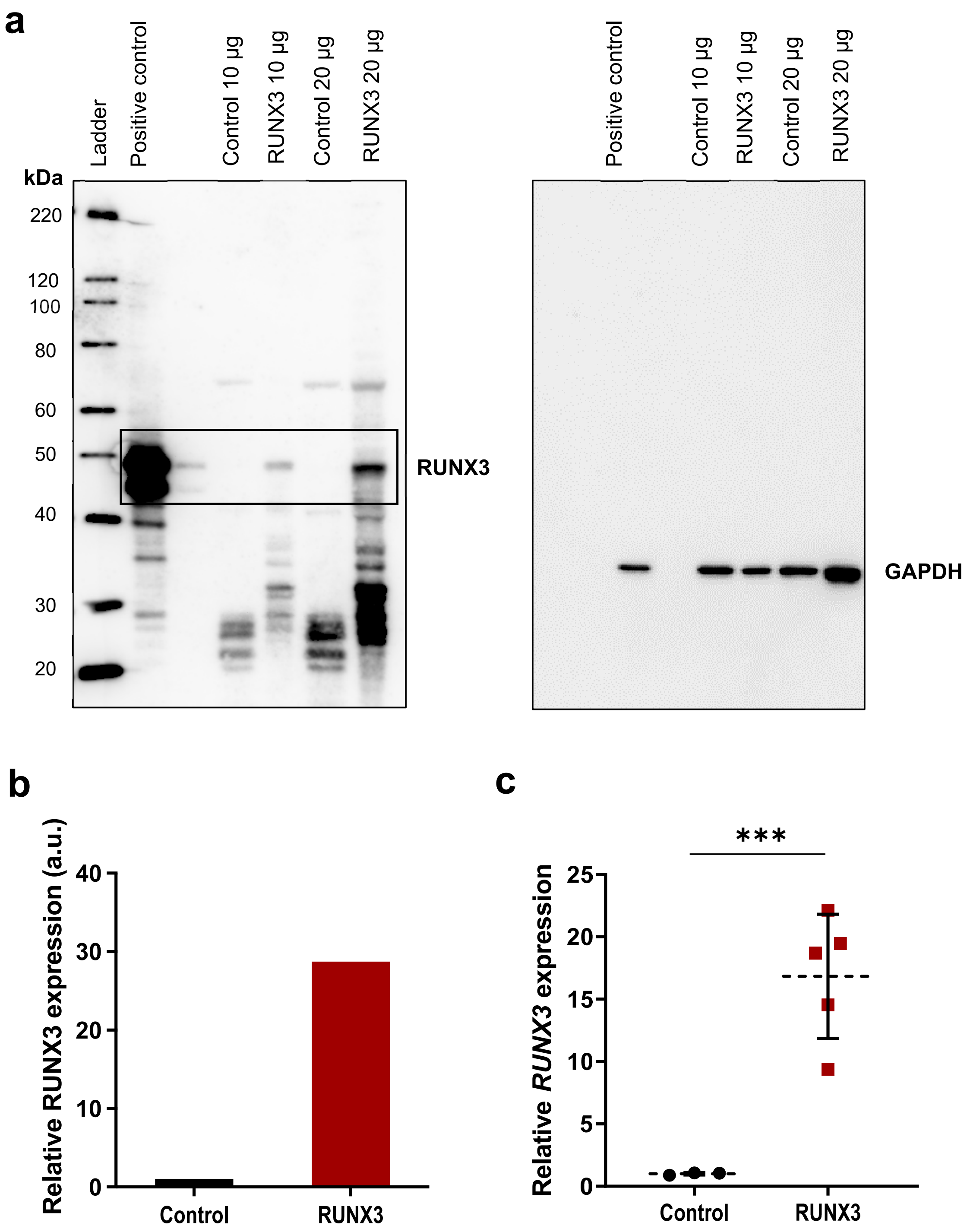

Supplemental Fig. S4. Validation of RUNX3 overexpression in human HSPC.

a (*Left panel):* Full length western blot showing RUNX3 total protein levels of control and RUNX3 CD34$^+$ HSPC (10 and 20 µg; day 6 of culture). Phoenix packaging cells overexpressing RUNX3 were used as a positive control. (*Right panel):* Full length western blot showing reprobing of the previous membrane for GAPDH, which was used as loading control. **b** Relative RUNX3 expression in control and RUNX3 HSPC cells normalized to control on day 6 of culture (n=1). A.u. – arbitrary units. **c** Relative *RUNX3* mRNA expression in control and RUNX3 GFP$^+$ cells on day 3 of culture. Data indicate mean ± 1SD (n≥3). GAPDH was used as endogenous control. Relative expression calculated using the comparative CT ($\Delta\Delta_{CT}$) method (see Methods). Significant difference of RUNX3-expressing cells from controls was analyzed by two-sample t-test, ****p<0.001*.

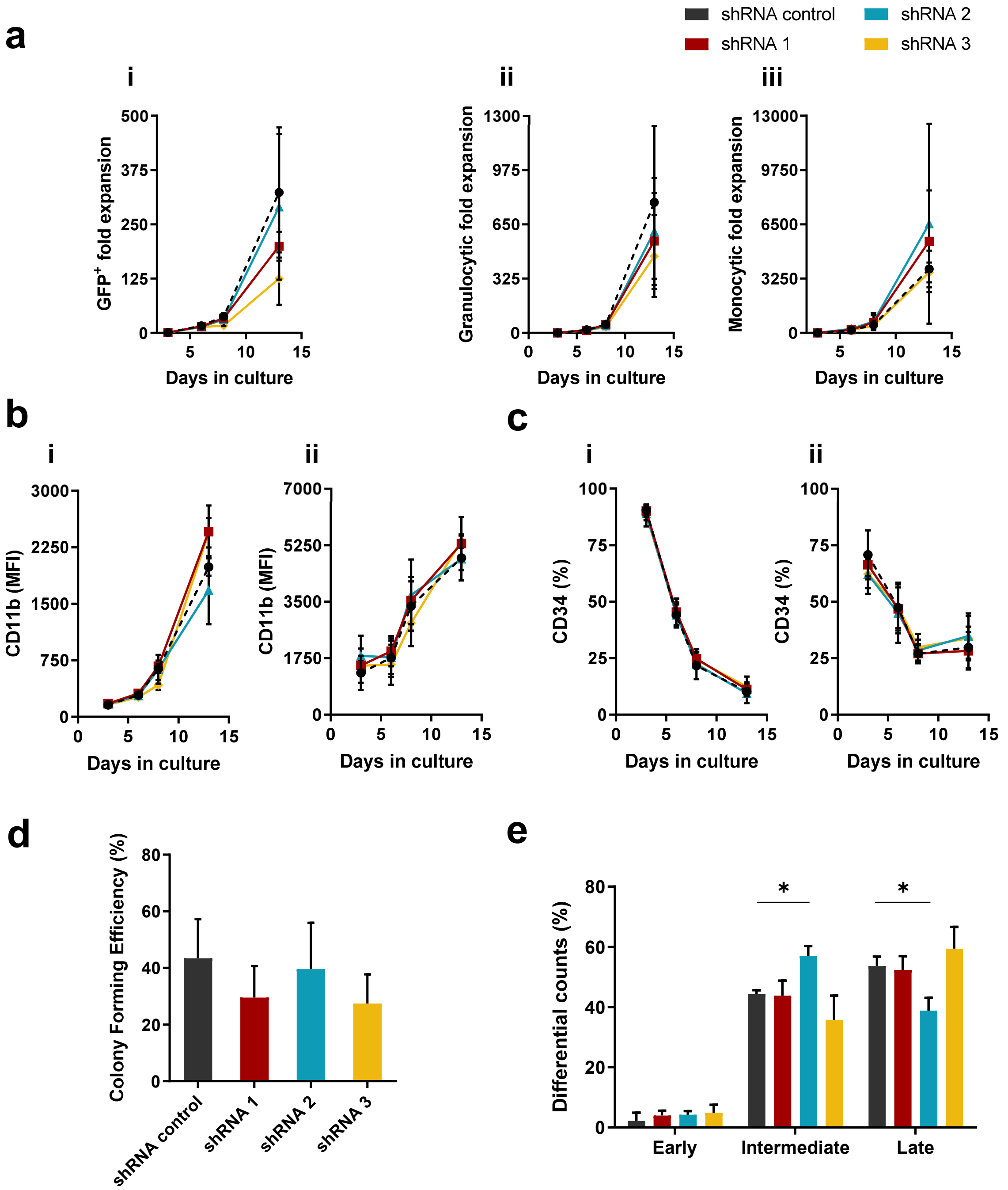

Supplemental Fig. S5. Effects of RUNX3 knockdown on myeloid development of human HSPC.

a Cumulative fold expansion of control and RUNX3 KD cultures in terms of GFP positivity (**i**), granulocytic committed cells (**ii**, $CD13^{-/+}CD36^{-}$), and monocytic committed cells (**iii**, $CD13^{+}CD36^{+}$) grown over 13 days in culture medium containing IL-3, SCF, G-CSF and GM-CSF. **b** Summary data of CD11b expression in terms of MFI in both control and RUNX3 KD cultures (**i**, granulocytic $CD13^{-/+}CD36^{-}$ cells; **ii**, monocytic $CD13^{+}CD36^{+}$ cells). **c** Summary data of CD34 percentage in both control and RUNX3 KD cultures ((**i**, granulocytic $CD13^{-/+}CD36^{-}$ cells; **ii**, monocytic $CD13^{+}CD36^{+}$ cells). **d** Colony forming efficiency of control and RUNX3 KD cultures after 7 days of growth in liquid culture containing IL-3 SCF, G-CSF and GM-CSF. **e** Differential counts of all cultures with morphology categorized into early (myeloblasts/promyelocytes), intermediate (myelocytes/metamyelocytes) and late phase (band/segmented granulocytic cells). Data indicate mean ± 1SD (n≥3). Statistical analysis was performed using ANOVA with Tukey's multiple comparisons test, *$p<0.05$ vs shRNA control.

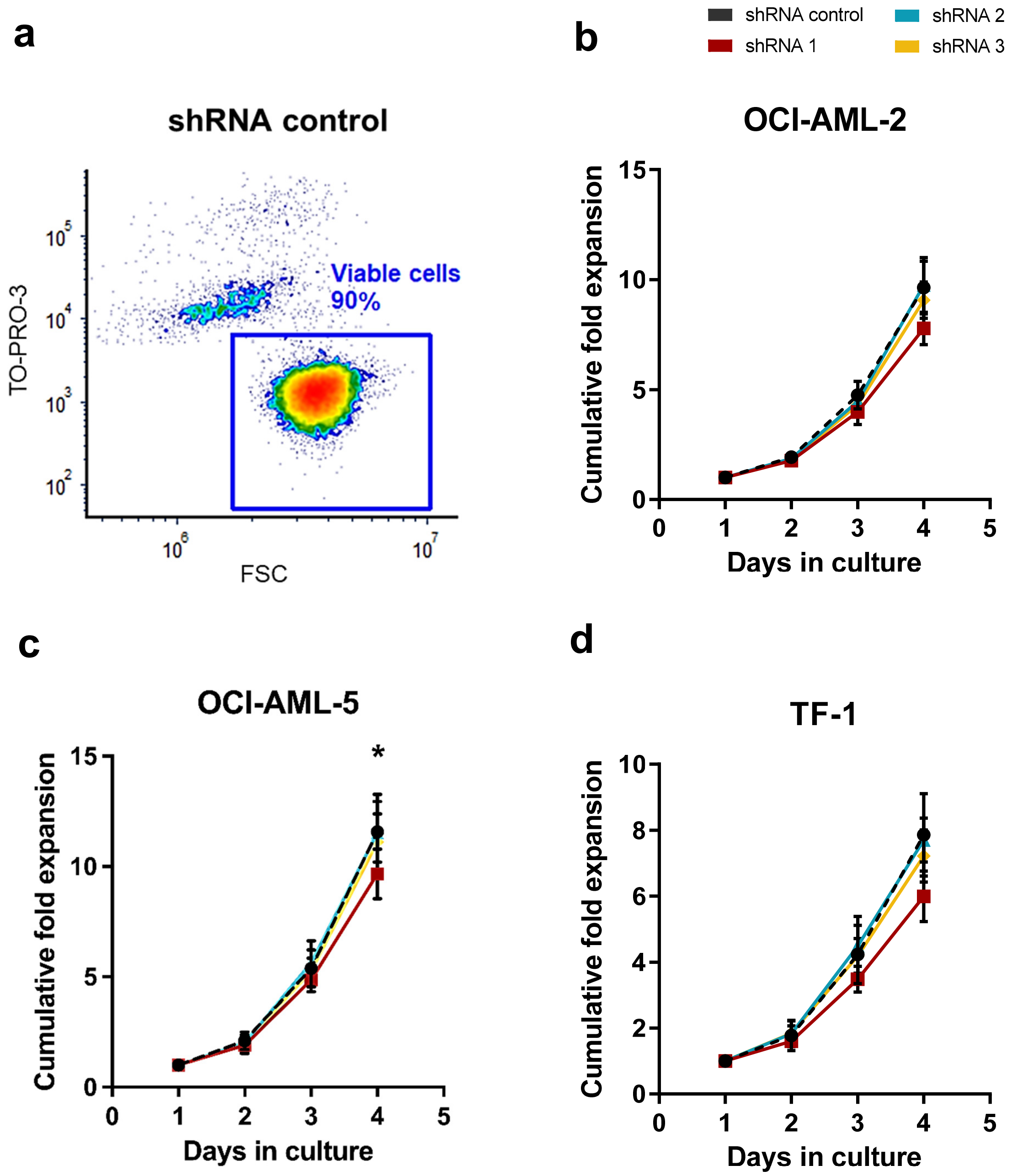

Supplemental Fig. S6. Effects of RUNX3 knockdown on AML cell line growth.

a Example density plot of shRNA control OCI-AML-2 culture labelled with the viability dye TO-PRO-3 (day 4). Debris were excluded from the analysis. **b** Cumulative fold expansion of puromycin-selected TF-1, OCI-AML-2, and OCI-AML-5 AML cells in liquid culture for control and RUNX3 shRNA cultures over 4 days of growth. Data indicate mean ± 1SD (n=3). Significant difference of shRNA control versus shRNA 1, 2 and 3 was analyzed by one-way ANOVA, Tuckey's test * $p<0.05$ vs shRNA1, shRNA 3.

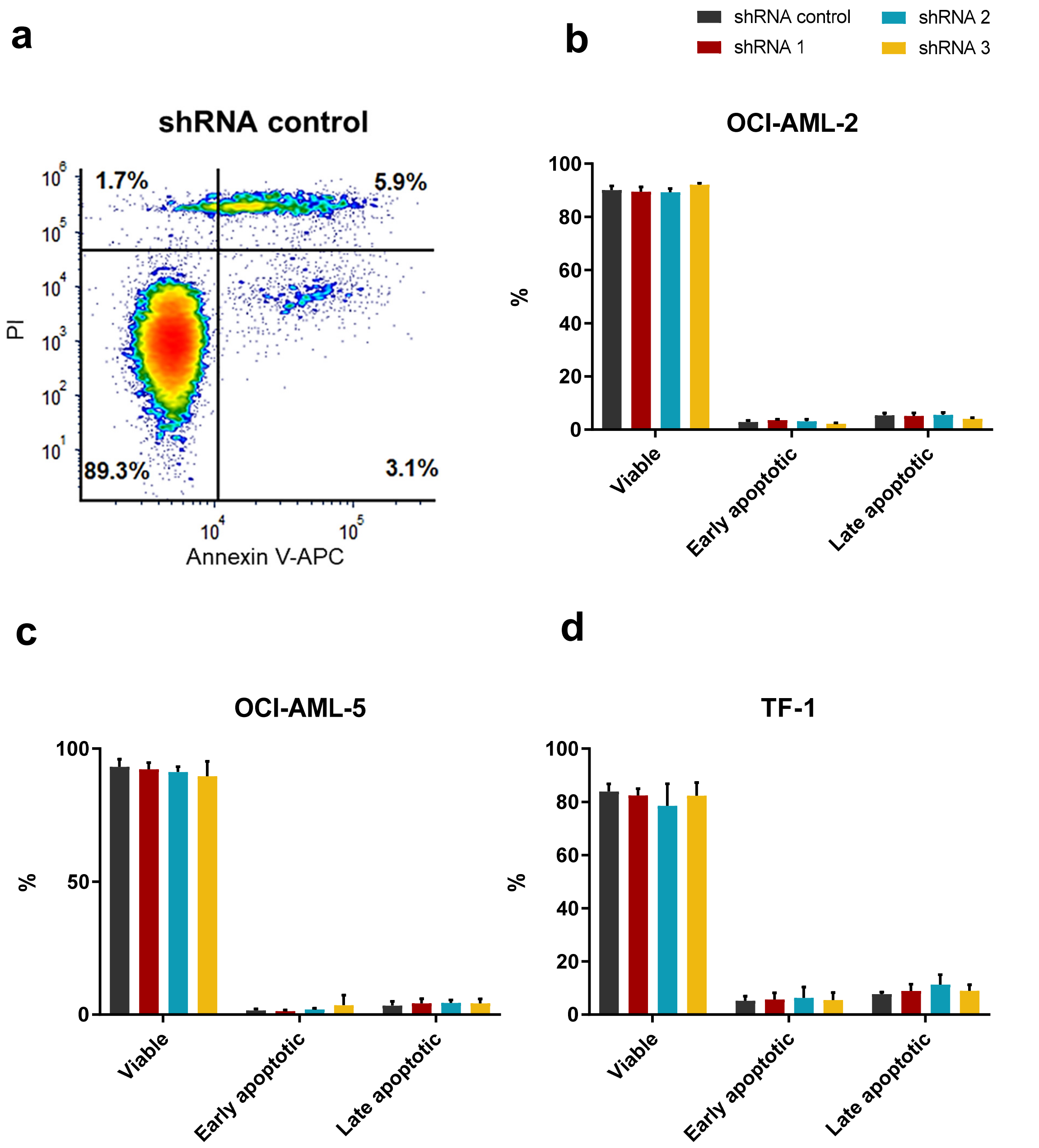

Supplemental Fig. S7. Effects of RUNX3 knockdown on apoptosis in AML cell lines.

a Example density plot of shRNA control OCI-AML-2 culture labelled with annexin V-APC and the viability dye PI (day 4). **b** Percentage of viable (PI$^-$Annexin V$^-$ cells), early apoptotic (PI$^-$Annexin V$^+$ cells), or late apoptotic cells (PI$^+$Annexin V$^+$ cells) following 4 days in culture. Data indicate mean ± 1SD (n=3).

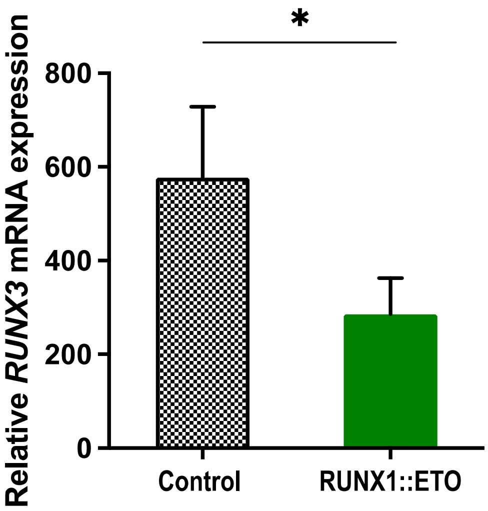

Supplemental Fig. S8. Expression of RUNX1::ETO as a single abnormality downregulates *RUNX3* mRNA expression in human HSPC.

Summary data of relative *RUNX3* mRNA expression in human cord blood-derived control and RUNX1::ETO expressing HSPC on day 3 of culture. Data obtained from E-MEXP-583 (Ref [4]). Data indicate mean ± 1SD (n=4). Significant difference of RUNX1::ETO expressing cells from controls was analyzed by paired t-test, *$p<0.05$.

a

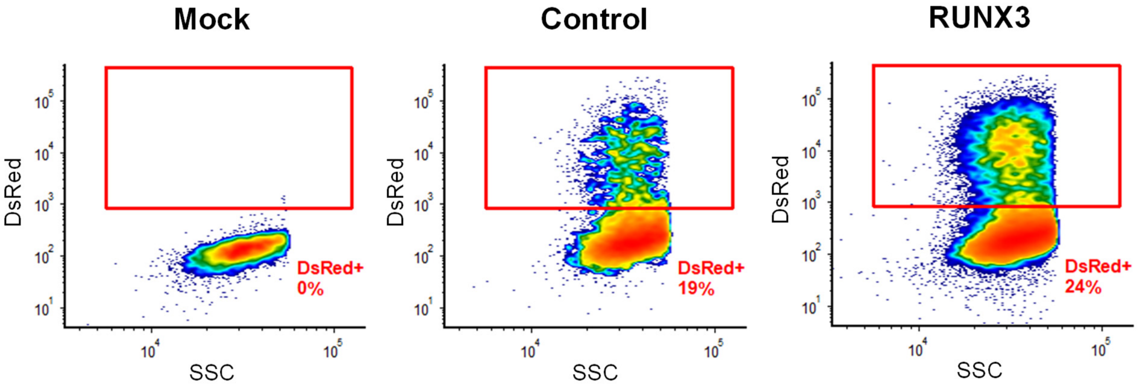

b

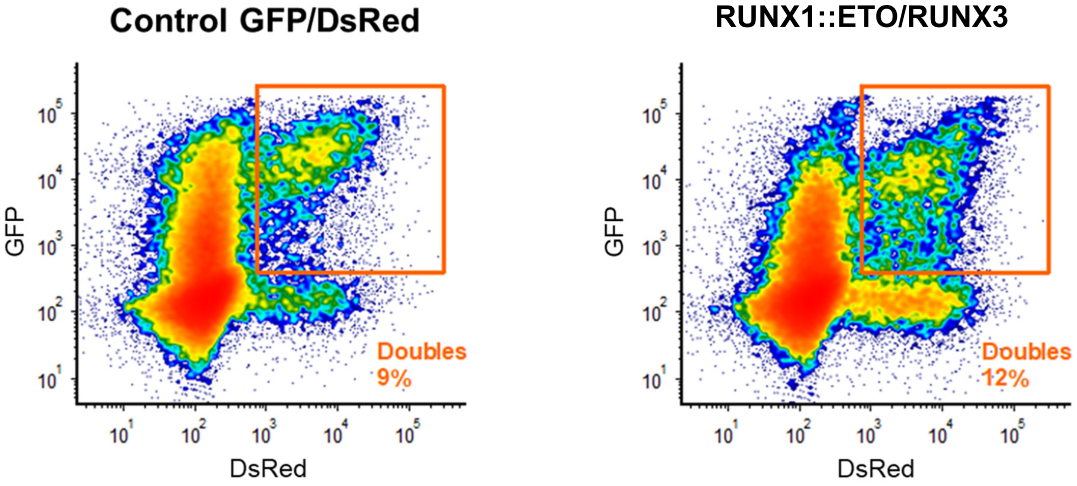

Supplemental Fig. S9. Gating strategy used for FACS of human HSPC single transduced with RUNX3 or double transduced with RUNX3 and RUNX1::ETO overexpression systems.

a Example density plots of control and RUNX3 cultures on day 3 before sorting for DsRed positivity. DsRed$^+$ gate delimits the sorted population and was defined using mock infected control cells (DsRed$^-$). Mock transduced cells were generated using the same infection approach and packaging cells supernatant with no retrovirus. **b** Example density plots of control GFP/DsRed and RUNX1::ETO/RUNX3 cultures on day 3 showing the double transduced HSPC populations. RUNX3 was overexpressed using a DsRed vector, whereas RUNX1::ETO was expressed using a GFP vector [2]. Doubles – gate used to analyze the double transduced (GFP$^+$DsRed$^+$) cells.

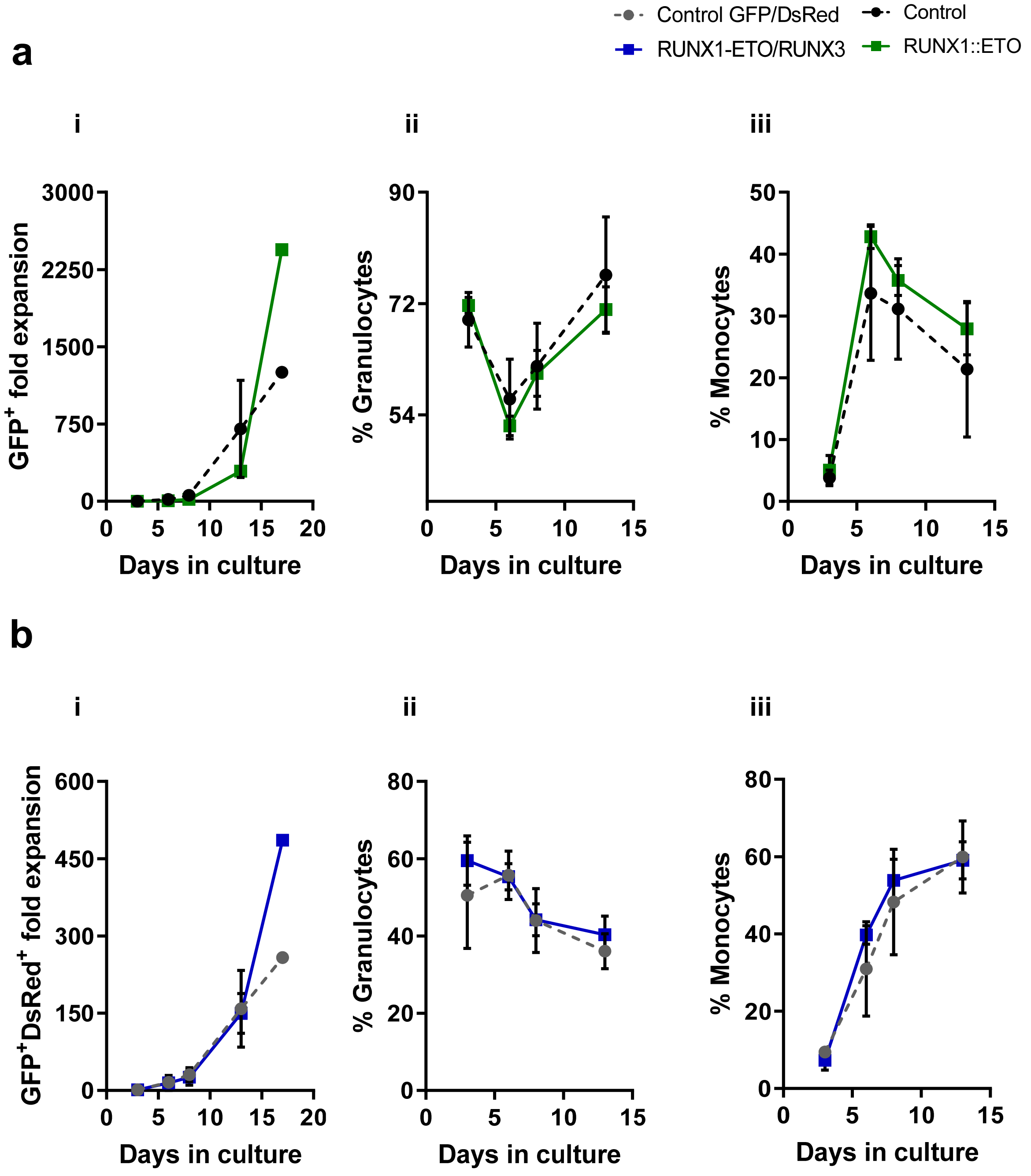

Supplemental Fig. S10. Expression of RUNX1::ETO in combination with RUNX3 does not impact the lineage balance during myeloid development of human HSPC.

a Summary data of control and RUNX1::ETO cultures in terms of cumulative fold expansion (**i**), percentage of granulocytic cells (**ii**, $CD13^{-/+}CD36^{-}$), and monocytic cells (**iii**, $CD13^{+}CD36^{+}$) during myeloid development over 13 days. **b** Summary data of $GFP^{+}DsRed^{+}$ control and RUNX1::ETO/RUNX3 cultures in terms of cumulative fold expansion (**i**), percentage of granulocytic cells (**ii**, $CD13^{-/+}CD36^{-}$), and monocytic cells (**iii**, $CD13^{+}CD36^{+}$) during myeloid development over 13 days. Data indicate mean ± 1SD (n≥3).

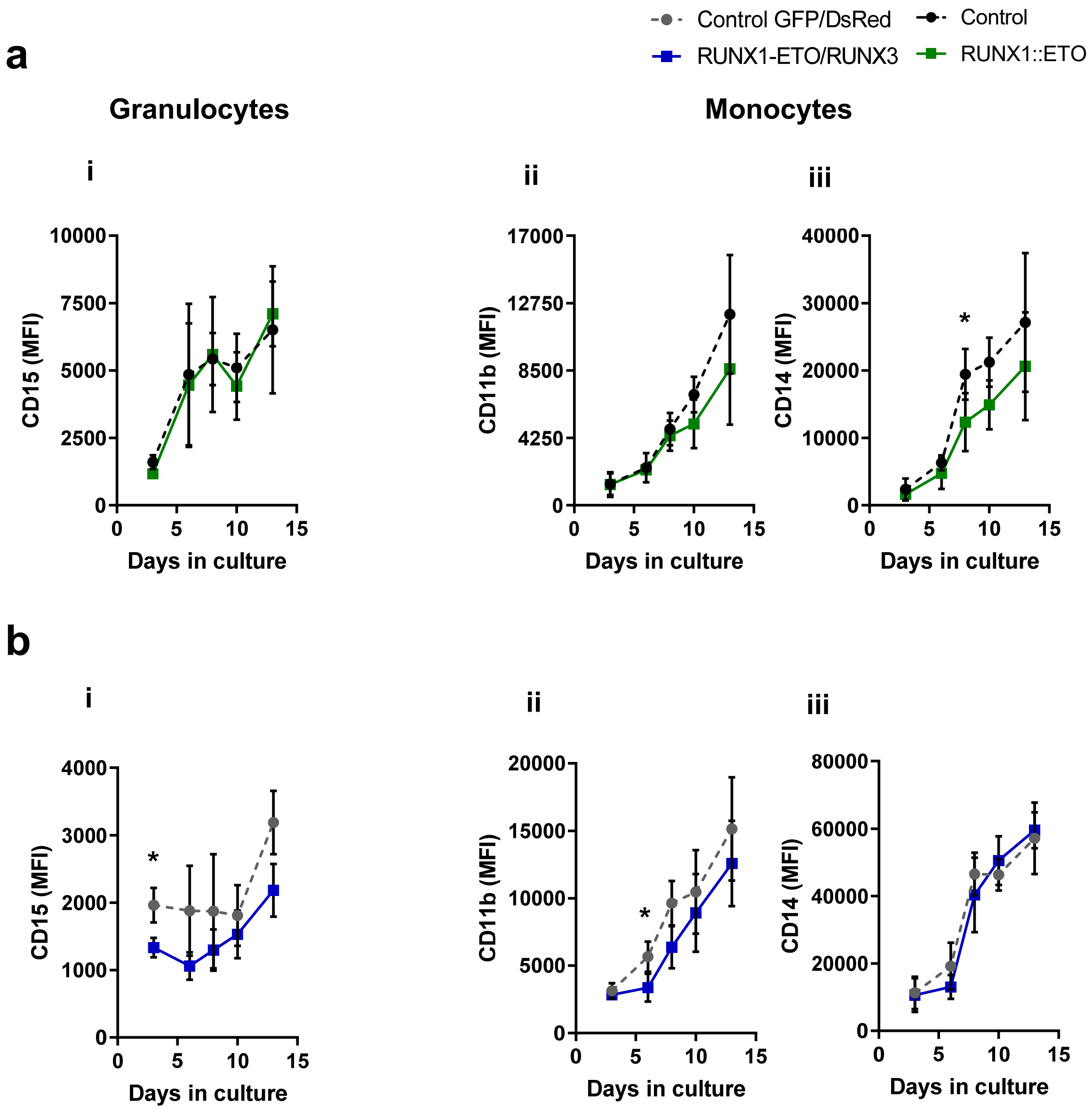

Supplemental Fig. S11. Abnormal granulocytic and monocytic development observed in cells expressing RUNX1::ETO in combination with RUNX3 overexpression.

a Summary data of control and RUNX1::ETO cultures during granulocytic development in terms of CD15 expression (**i**) in MFI, and monocytic development in terms of CD11b (**ii**) and CD14 (**iii**) expression in MFI over 13 days. Data indicate mean ± 1SD (n=3). Significant difference of RUNX1::ETO cells vs GFP^+ control was analyzed by paired t-test, *$p<0.05$. **b** Summary data of GFP^+DsRed^+ control and RUNX1::ETO/RUNX3 cultures during granulocytic development in terms of CD15 expression (**i**) in MFI, and monocytic development in terms of CD11b (**ii**) and CD14 (**iii**) expression in MFI over 13 days. Data indicate mean ± 1SD (n=3). Significant difference of RUNX1::ETO/RUNX3 cells vs GFP/DsRed control was analyzed by paired t-test, *$p<0.05$.

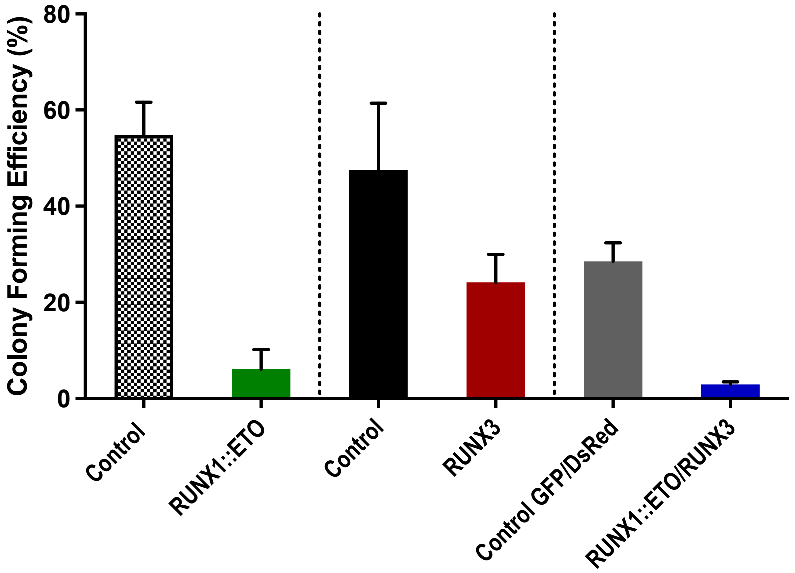

Supplemental Fig. S12. Expression of RUNX1::ETO in combination with RUNX3 inhibits the colony formation ability of human erythroid progenitors.

Summary data of erythroid colony forming efficiency for control and RUNX1::ETO cells, as well as control and RUNX3 cells, and control GFP/DsRed and RUNX1::ETO/RUNX3 cells following 7 days of growth in liquid culture containing IL-3, SCF, IL-6 and EPO. Data indicate mean ± 1SD (n=2). On day 3 of culture, single transduced RUNX1::ETO cells and respective control were sorted for GFP positivity and enriched for primitive erythroid cells ($CD13^{-}$), similarly RUNX3 cells and respective controls were sorted for DsRed positivity/$CD13^{-}$; double cultures were sorted for GFP/DsRed /$CD13^{-}$.

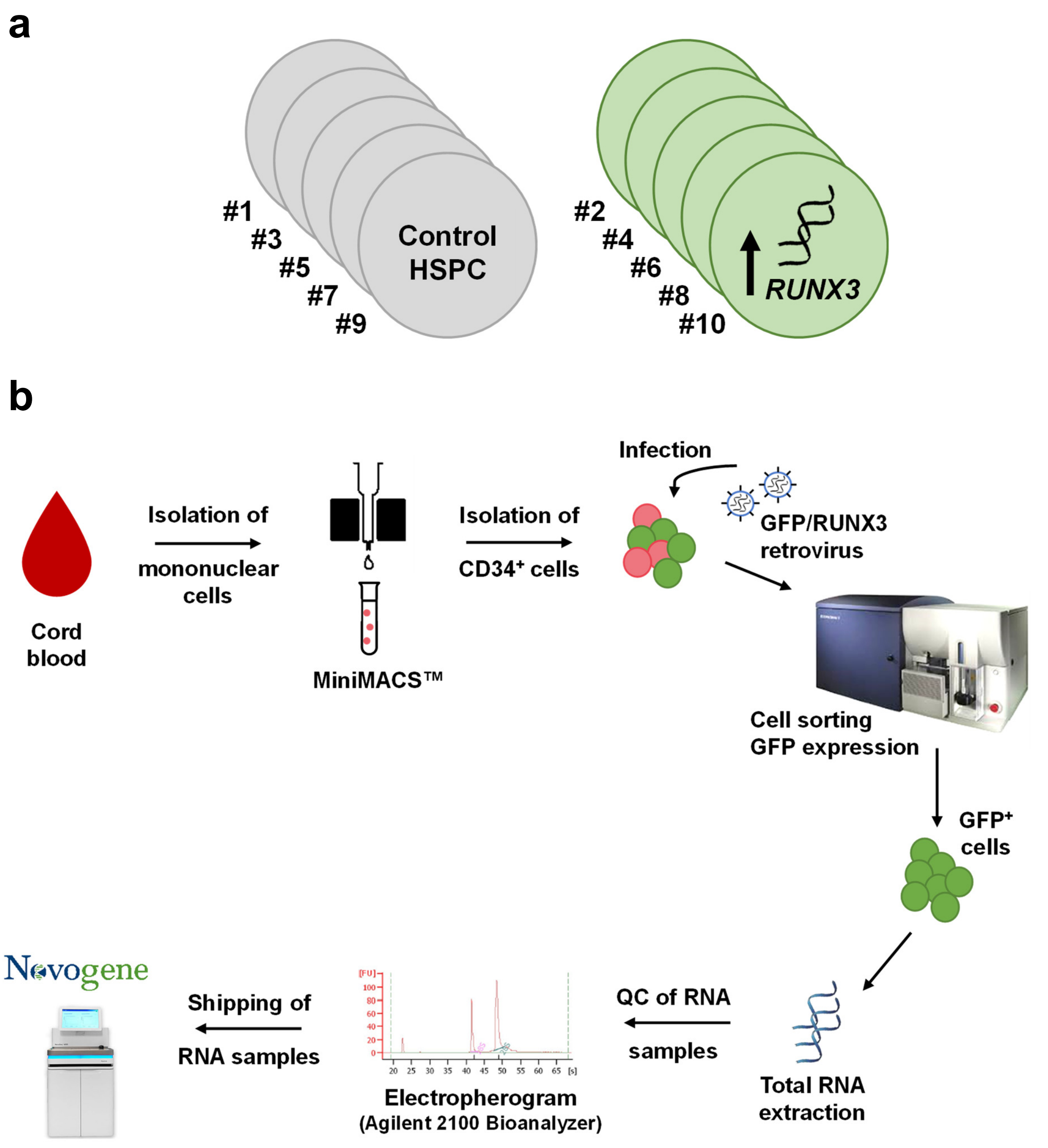

Supplemental Fig. S13. Experimental design of RNA-seq analysis of transcriptional changes associated with RUNX3 overexpression in HSPC.

a Control and RUNX3 HSPC (5 independent experiments; samples #1 to #10) were analyzed by RNA-seq as in b. **b** CD34$^+$ HSPC were isolated using the MiniMACS™ magnetic sorting system for CD34 positivity and subsequently infected with control (PINCO GFP) or RUNX3 GFP retrovirus. Following enrichment for GFP expression by FACS, total RNA was extracted using the RNeasy Plus Mini Kit and RNA integrity number (RIN) was assessed using Agilent 2100 Bioanalyzer. Samples with a RIN above 8 were used for RNA-seq.

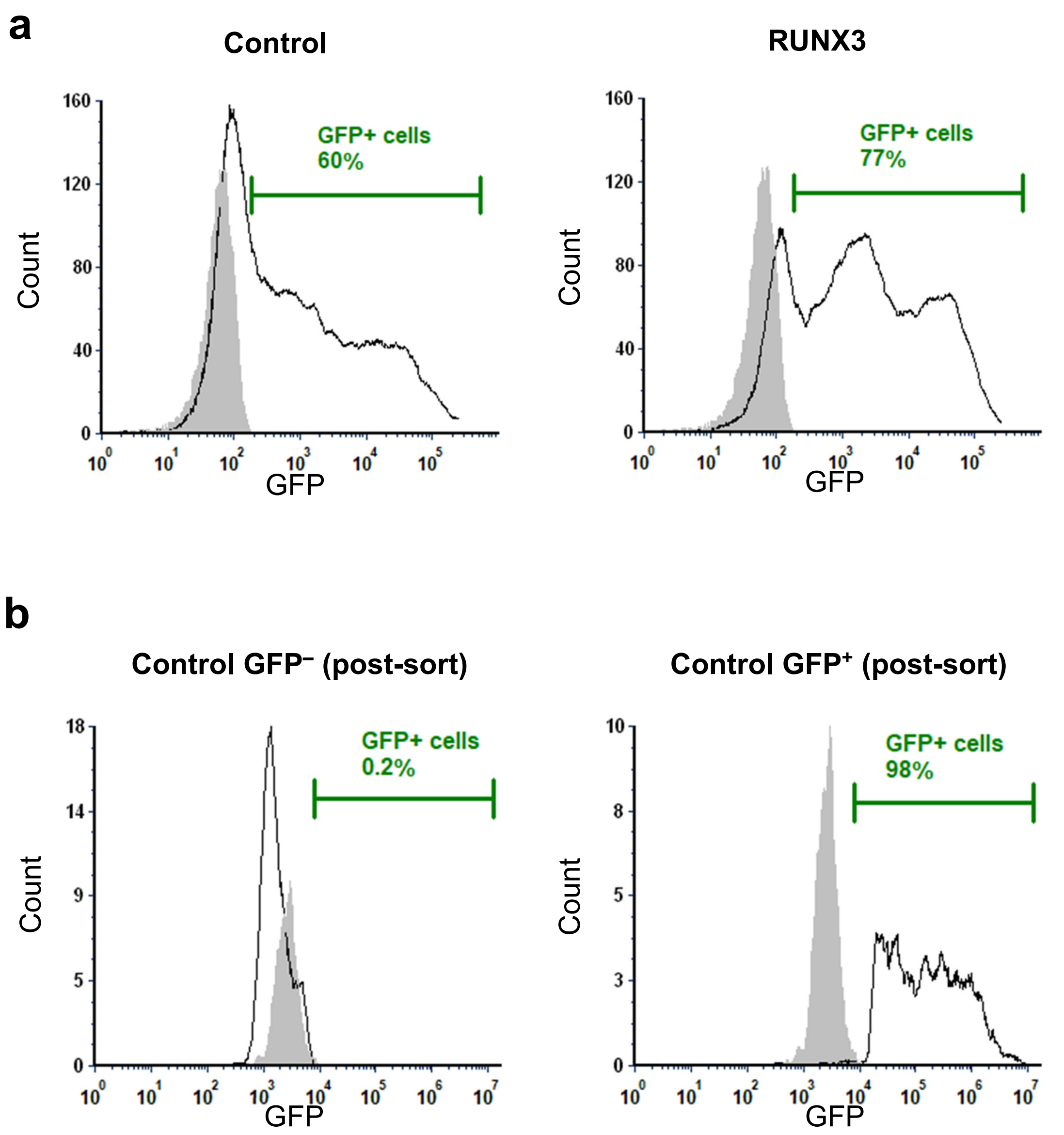

Supplemental Fig. S14. Successful expression and enrichment of GFP in human HSPC.

a Representative flow cytometric histograms of GFP expression for control and RUNX3 infected cells (pre-sorted cultures day 3). GFP^+ gate delimits the sorted population and was defined using mock infected control cells (GFP^-). Data obtained using BD FACSAria™ II cytometer. **b** Representative histograms for control culture post-sorting for GFP^- and GFP^+ cells. Data obtained using BD Accuri™ C6 Plus cytometer. Mock HSPC – gray; Transduced HSPC – black.

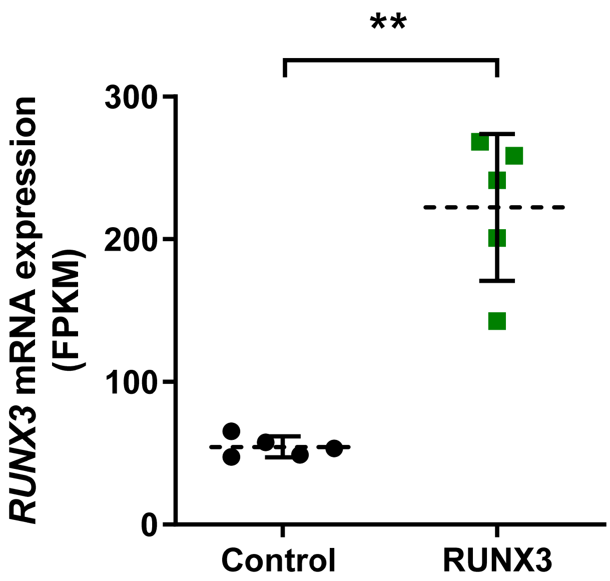

Supplemental Fig. S15. *RUNX3* mRNA is successfully overexpressed in human HSPC.

Summary data of *RUNX3* mRNA expression (Fragments per kilobase of transcript per million, FPKM) for control and RUNX3 overexpressing HSPC obtained using the HTSeq package. Dotted line indicates mean ± 1SD (n=5). Significant difference of RUNX3 overexpressing cells compared to controls was analyzed by paired t-test, ***$p<0.01$.*

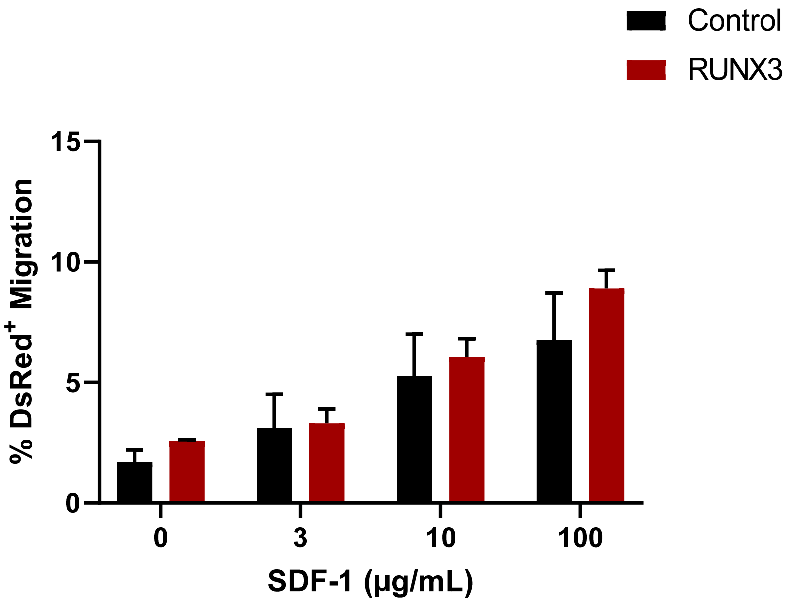

Supplemental Fig. S16. Effects of RUNX3 overexpression on human HSPC migration towards an SDF-1 gradient.

Summary data of the percentage of migration for control and RUNX3 DsRed$^+$ HSPC (day 6) in response to SDF-1 exposure. Data indicates mean ± 1SD (n=3).

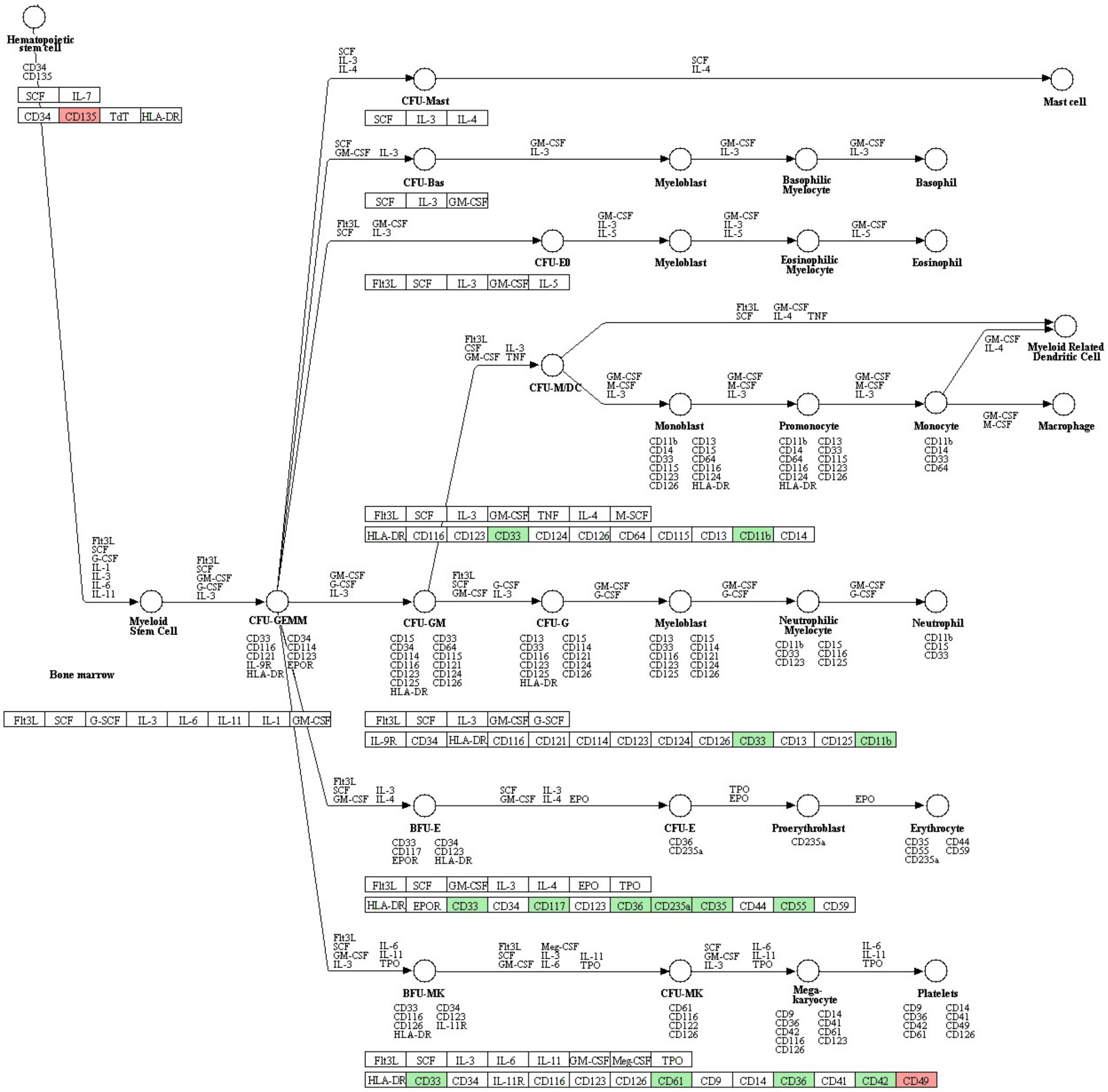

Supplemental Fig. S17. RUNX3 overexpression dysregulates important genes involved in human hematopoiesis.

Representation of the human Hematopoietic Cell Lineage KEGG pathway map (hsa04640). Cellular stages are identified by the specific expression of genes, which are highlighted in red for upregulation and green for downregulation associated with RUNX3 overexpression in HSPC. The lymphoid arm of hematopoiesis was removed from this pathway. Relevant cytokines involved in hematopoiesis are represented in each developmental stage. Important receptors and cytokines involved in hematopoiesis are represented in boxes and summarized under each developmental pathway/lineage.

References

1. Grignani F, Kinsella T, Mencarelli A, Valtieri M, Riganelli D, Grignani F*, et al.* High-efficiency gene transfer and selection of human hematopoietic progenitor cells with a hybrid EBV/retroviral vector expressing the green fluorescence protein. *Cancer Res*. 1998; **58**(1): 14-19.

2. Tonks A, Pearn L, Tonks AJ, Pearce L, Hoy T, Phillips S*, et al.* The AML1-ETO fusion gene promotes extensive self-renewal of human primary erythroid cells. *Blood*. 2003; **101**(2): 624-632.

3. Tonks A, Tonks AJ, Pearn L, Mohamad Z, Burnett AK, Darley RL. Optimized retroviral transduction protocol which preserves the primitive subpopulation of human hematopoietic cells. *Biotechnol Prog*. 2005; **21**(3): 953-958.

4. Tonks A, Pearn L, Musson M, Gilkes A, Mills KI, Burnett AK*, et al.* Transcriptional dysregulation mediated by RUNX1-RUNX1T1 in normal human progenitor cells and in acute myeloid leukaemia. *Leukemia*. 2007; **21**(12): 2495-2505.

5. Justus CR, Leffler N, Ruiz-Echevarria M, Yang LV. In vitro cell migration and invasion assays. *J Vis Exp*. 2014; (88).

6. Rapin N, Bagger FO, Jendholm J, Mora-Jensen H, Krogh A, Kohlmann A*, et al.* Comparing cancer vs normal gene expression profiles identifies new disease entities and common transcriptional programs in AML patients. *Blood*. 2014; **123**(6): 894-904.

7. Svendsen JB, Baslund B, Cramer EP, Rapin N, Borregaard N, Cowland JB. MicroRNA-941 Expression in Polymorphonuclear Granulocytes Is Not Related to Granulomatosis with Polyangiitis. *PLoS One*. 2016; **11**(10): e0164985.

8. Cancer Genome Atlas Research N, Ley TJ, Miller C, Ding L, Raphael BJ, Mungall AJ*, et al.* Genomic and epigenomic landscapes of adult de novo acute myeloid leukemia. *N Engl J Med*. 2013; **368**(22): 2059-2074.

9. Cerami E, Gao J, Dogrusoz U, Gross BE, Sumer SO, Aksoy BA*, et al.* The cBio cancer genomics portal: an open platform for exploring multidimensional cancer genomics data. *Cancer Discov*. 2012; **2**(5): 401-404.

10. Gao J, Aksoy BA, Dogrusoz U, Dresdner G, Gross B, Sumer SO*, et al.* Integrative analysis of complex cancer genomics and clinical profiles using the cBioPortal. *Sci Signal*. 2013; **6**(269): pl1.
